# Evolution of the Subgroup 6 *R2R3-MYB* Genes and Their Contribution to Floral Color in the Perianth-Bearing Piperales

**DOI:** 10.3389/fpls.2021.633227

**Published:** 2021-04-09

**Authors:** Sarita Muñoz-Gómez, Harold Suárez-Baron, Juan F. Alzate, Favio González, Natalia Pabón-Mora

**Affiliations:** ^1^Facultad de Ciencias Exactas y Naturales, Instituto de Biología, Universidad de Antioquia, Medellín, Colombia; ^2^Centro Nacional de Secuenciación Genómica – CNSG, Sede de Investigación Universitaria, Departamento de Microbiología y Parasitología, Facultad de Medicina, Universidad de Antioquia, Medellín, Colombia; ^3^Universidad Nacional de Colombia, Sede Bogotá, Facultad de Ciencias, Instituto de Ciencias Naturales, Bogotá, Colombia

**Keywords:** anthocyanins, Aristolochiaceae, *Asarum*, flavonoids, floral color, petaloid sepals, *Saruma*, subgroup 6 *R2R3-MYB* genes

## Abstract

Flavonoids, carotenoids, betalains, and chlorophylls are the plant pigments responsible for floral color. Anthocyanins, a class of flavonoids, are largely responsible for the red, purple, pink, and blue colors. *R2R3-MYB* genes belonging to subgroup 6 (SG6) are the upstream regulatory factors of the anthocyanin biosynthetic pathway. The canonical members of these genes in *Arabidopsis* include *AtMYB75*, *AtMYB90*, *AtMYB113*, and *AtMYB114*. The Aristolochiaceae is an angiosperm lineage with diverse floral groundplans and perianth colors. *Saruma henryi* exhibits a biseriate perianth with green sepals and yellow petals. All other genera have sepals only, with colors ranging from green (in *Lactoris*) to a plethora of yellow to red and purple mixtures. Here, we isolated and reconstructed the SG6 *R2R3-MYB* gene lineage evolution in angiosperms with sampling emphasis in Aristolochiaceae. We found numerous species-specific duplications of this gene lineage in core eudicots and local duplications in Aristolochiaceae for *Saruma* and *Asarum*. Expression of SG6 *R2R3-MYB* genes examined in different developmental stages and plant organs of four Aristolochiaceae species, largely overlaps with red and purple pigments, suggesting a role in anthocyanin and flavonoid synthesis and accumulation. A directed RNA-seq analysis corroborated our RT-PCR analyses, by showing that these structural enzymes activate during perianth development in *Aristolochia fimbriata* and that the regulatory genes are expressed in correlation with color phenotype. Finally, the reconstruction of the flavonoid and anthocyanin metabolic pathways using predicted peptides from transcriptomic data show that all pivotal enzymes are present in the analyzed species. We conclude that the regulatory genes as well as the biosynthetic pathway are largely conserved across angiosperms. In addition, the Aristolochiaceae emerges as a remarkable group to study the genetic regulatory network for floral color, as their members exhibit an outstanding floral diversity with elaborate color patterns and the genetic complement for SG6 *R2R3-MYB* genes is simpler than in core eudicot model species.

## Introduction

The floral color palette is remarkable across angiosperms and can be linked to pollinator attraction during anthesis playing a key role in reproduction (Frey et al., 2011). Although all floral parts accumulate pigments, color display shifts during development are striking in the perianth. The chlorophylls, the carotenoids, the betalains, and the flavonoids are the primary plant pigments synthesized. Under different environmental and developmental conditions, these can be found in both vegetative and reproductive plant organs. The chlorophylls are produced in the chloroplast, are green and are required for photosynthesis, but are not leaf exclusive and can be present in the flowers (Narbona et al., 2014). The carotenoids are lipid-soluble molecules also produced in the chloroplast, which are responsible for the yellow, red, and orange colors, and are involved in the maintenance of the photosynthetic apparatus integrity (Tanaka et al., 2008; Chatham et al., 2019). On the other hand, betalains and flavonoids are both water-soluble molecules. Betalains are only present in members of the order Caryophyllales and are responsible for red and violet to yellow and orange hues. Betalains and anthocyanins are mutually exclusive since they do not occur simultaneously in any plant (Clement et al., 1994). There are many types of flavonoids such as flavonols, flavones, anthocyanins, and proanthocyanidins (Matsui and Walker, 2019). Flavonoids, in general, play various roles including the attraction of pollinators, the quenching of UV light (Tanaka et al., 2008) and protection against stress (Winkel-Shirley, 2001, 2002), such as resistance to aluminum toxicity (Kidd et al., 2001) or stress response by stomatal opening control *via* auxins (Dietrich et al., 2001). In particular, anthocyanins are responsible for colors such as red, blue, purple, and pink in different plant structures, but especially in floral organs (Chatham et al., 2019). In flowers, anthocyanins are the primary attractants for insects, birds and other biotic vectors aiding in reproduction (Jackman and Smith, 1996; Kong et al., 2003). Similarly, anthocyanin-derived fruit color can increase frugivory and seed dispersal. Anthocyanins are glycosides of anthocyanidins (Holton and Cornish, 1995), and their colors can change according to pH, as red tones prevail in acidic conditions whereas blue tones are characteristic of alkaline conditions (Calderaro et al., 2020). The most common anthocyanidins found in anthocyanins are cyanidin, delphinidin, pelargonidin, peonidin, petunidin, and malvidin (Clifford, 2000). Anthocyanins are made from a C6-C3-C6 skeleton from which different modifications occur. Both, the type and the color of the resulting anthocyanin depend primarily on the addition of methoxyl or hydroxyl groups to the B ring (Chatham et al., 2019).

As many other metabolic plant routes, the flavonoid biosynthetic pathway, responsible for anthocyanidin production, is regulated at the transcriptional level. A MBW (MYB-bHLH-WD) transcriptional activation complex controls the latter steps of the flavonoid pathway (Schwinn et al., 2016). This complex is the result of the physical interaction between a MYB transcription factor, a basic Helix-Loop-Helix (bHLH) factor and a WD40-repeat protein (González et al., 2008; Zhang and Schrader, 2017). Here we largely concentrate on the *MYB* genes, which are a large family of transcription factors (TFs) found in most eukaryotes (Klempnauer et al., 1982). In plants, *MYB* genes are divided into four types: *4R-MYB*, *3R-MYB*, *“MYB-related,”* and the *R2R3-MYB*. The *R2R3-MYB* clade is the largest and most functionally diverse type, as these genes control the production of primary and secondary metabolites and the specialization of epidermal cells as trichomes or root hairs, among other functions (Higginson et al., 2003; Tominaga et al., 2008). The *R2R3-MYB* TFs can act as transcriptional activators (Noda et al., 1994) or repressors (Park et al., 2008). The classification of *R2R3-MYB* genes comes primarily from *Arabidopsis thaliana* homologs (Kranz et al., 1998; Romero et al., 1998; Stracke et al., 2001). Stracke et al. (2001), designated subgroups based on 125 sequences, using function and specific motifs outside the MYB domain as main classifiers. The R2R3-MYB proteins receive their name based on the R2 and R3 domains found in the N-terminal portion of the protein. Each domain contains around 53 amino acids and forms three alpha-helices with the help of three spaced tryptophan residues that form a hydrophobic cluster (Stracke et al., 2001). *R2R3-MYB* genes, known to play roles in the early stages of the anthocyanin biosynthesis pathway, have been assigned to Subgroup 6 (hereafter referred to as SG6), which include the *Arabidopsis* paralogs *AtMYB75*, *AtMYB90*, *AtMYB113*, and *AtMYB114*.

The recruitment of *R2R3-MYB* gene homologs in pigmentation has been studied in *Antirrhinum*, *Clarkia*, *Iochroma*, *Ipomoea*, *Lilium*, *Mimulus*, *Oenanthe*, *Petunia*, *Phalaenopsis*, and various Rosaceae (Bradshaw et al., 1995; Quattrocchio et al., 1999; Zufall and Rausher, 2003; Morita et al., 2006; Schwinn et al., 2006; Lin-Wang et al., 2010; Shang et al., 2011; Lowry et al., 2012; Wu et al., 2013; Hsu et al., 2015; Pérez-Díaz et al., 2016; Yamagishi, 2016; Feng et al., 2018; Gates et al., 2018; Ding et al., 2020; Lin and Rausher, 2020). In contrast, no studies are available linking *R2R3-MYB* homologs to color production in early divergent angiosperms. The only available study by Zhang et al. (2020) in Nymphaeaceae is concentrated in the flavonoid biosynthetic pathway in *Nymphaea colorata* and *N. nucifera*. In these species, the flavonoid pathway enzymes are more expressed in blue petals compared to white petals, and very specific delphinidin molecules [delphinidin 3′-O-(2″-O-galloyl-6″-O-acetyl-B-galactopyranoside)] have been found in the blue petals of *N. colorata* (Zhang et al., 2020).

**FIGURE 1:**
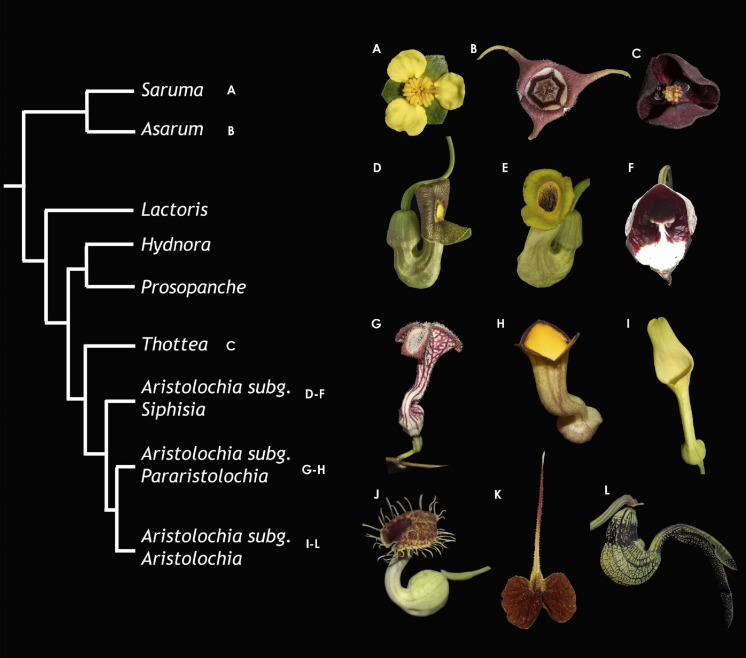
Summarized phylogeny of the Aristolochiaceae with representative photographs for the main lineages. **(A)** *Saruma henryi*. **(B)** *Asarum canadense*. **(C)** *Thottea siliquosa*. **(D)** *Aristolochia macrophylla*. **(E)** *A. manshuriensis*. **(F)** *A. arborea*. **(G)** *A. deltantha*. **(H)** *A. praevenosa*. **(I)** *A. clematitis*. **(J)** *A. fimbriata*. **(K)** *A. lindneri*. **(L)** *A. ringens*.

The Aristolochiaceae includes all perianth-bearing Piperales, namely, *Aristolochia, Asarum, Hydnora, Lactoris, Prosopanche, Thottea*, and *Saruma* (Wanke et al., 2006; Figure 1). The monotypic *Saruma* is the only genus having a biseriate perianth formed by three green sepals and three yellow petals (González and Stevenson, 2000; Pabón-Mora et al., 2020). The remaining genera, *Aristolochia*, *Asarum, Hydnora, Lactoris, Prosopanche*, and *Thottea* have three sepals partially or totally fused with each other, exhibiting different colors (Pabón-Mora et al., 2020). In *Asarum*, sepals are usually dark purple but some species exhibiting yellow sepals can be found. Sepals in *Hydnora* and *Prosopanche*, the only holoparasitic members of the family, exhibit color ranges from dark purple and bright red or orange tones to white or cream (Musselman and Visser, 1989). The three sepals in the sole species of *Lactoris (L. fernandeziana)* are light-green (Bernardello et al., 1999; González and Rudall, 2001). Perianth colors in *Thottea* vary from dark purple to brown to red, suffused with yellow or white (Shaiju and Omanakumari, 2010). The highly elaborated, sepal-derived perianth in *Aristolochia*, is exceedingly diverse in terms of color, size, and shape (Wagner et al., 2012, 2014; Pabón-Mora et al., 2020; Figure 1). *Aristolochia* sepals are often green in their outer surface and variously colored with purple, red, yellow, or white in their inner surface. Because typical petal features such as colors (other than green), papillae and osmophores are present in *Aristolochia* sepals, these organs are thought to present a transfer of function from petals (Pabón-Mora et al., 2015). Additionally, different colors are displayed forming a vast range of species-specific patterns, including reticulations, streaks, lines, spots or dots that are showier in the inner surface of the limb.

Here we aim to: (1) identify SG6 *R2R3-MYB* homologs, putatively associated with flavonoid production in members of the Aristolochiaceae; (2) reconstruct their evolutionary history across flowering plants, with a sampling focused on non-core eudicots; (3) compare their expression during flower development on selected genera within the Aristolochiaceae; (4) understand the spatio-temporal activation of TFs and enzymes during the development of *Aristolochia fimbriata*; and (5) plot the flavonoid and anthocyanin metabolic pathways for representative members of the Aristolochiaceae.

## Materials and Methods

### Isolation and Phylogenetic Analyses of SG6 *R2R3-MYB* Genes

Canonical SG6 *R2R3-MYB* genes from *Arabidopsis thaliana* (*AtMYB75*, *AtMYB90*, *AtMYB113*, and *AtMYB114*) were used as queries to identify putative homologs in the available Aristolochiaceae transcriptomes (see Pabón-Mora et al., 2015, 2020). The transcriptomes were generated from mixed leaves, flowers, and fruits (when available) for the following species: *Aristolochia arborea, A. clematitis*, *A. deltantha*, *A. lindneri*, *A. macrophylla*, *A. manshuriensis, A. praevenosa*, *A. ringens, Asarum canadense*, *A. europaeum*, *Saruma henryi*, and *Thottea siliquosa*. The *Arabidopsis thaliana* sequences were retrieved from TAIR^1^. All other sequences included in the analysis outside Aristolochiaceae and Brassicaceae were downloaded from public sequence repositories such as NCBI^2^, Phytozome^3^, OneKP (Leebens-Mack et al., 2019)^4^, The Plant Transcription Factor Database (Jin et al., 2016)^5^, and our own transcriptomes. Searches were performed using BLASTN (Altschul et al., 1990). Closely related *Arabidopsis R2R3-MYB* subgroups SG4, SG5, SG7, and SG15, were also retrieved and used as outgroups. The selection of several subgroups as outgroups allowed us to identify SG6 orthologs. Homologs included in these analyses are in Supplementary Table 1. Newly isolated sequences from this work can be found in GenBank under the accessions MW125647–MW125662 and MW788582–MW788641.

**FIGURE 2:**
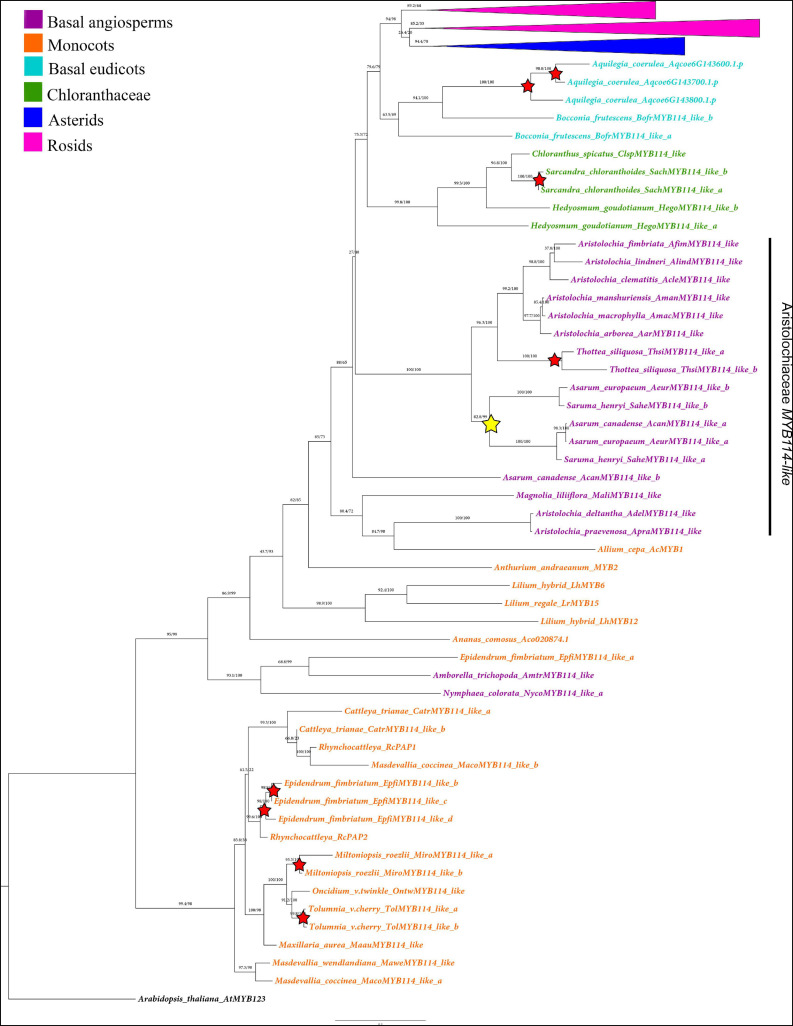
Maximum likelihood analysis of the SG6 *R2R3-MYB* genes with expanded view of the non-core eudicot gene homologs. Yellow star indicates large-scale duplication events in the Aristolochiaceae prior to the diversification of *Asarum* and *Saruma*. Red stars represent species-specific duplication events. Color clades follow the conventions in the top left.

**FIGURE 3:**
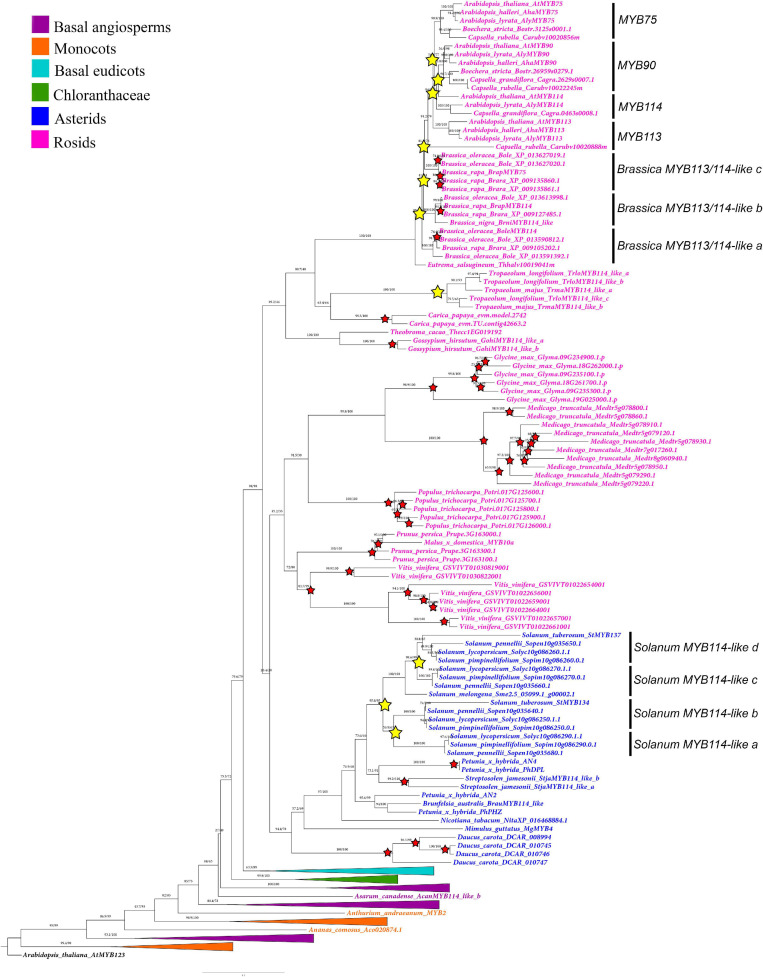
Maximum likelihood analysis of the SG6 *R2R3-MYB* genes with expanded view of the core-eudicot gene homologs. Yellow stars indicate large-scale duplication events in *Solanum* and Brassicales. Red stars represent species-specific duplication events. Color clades follow the conventions in the top left.

All sequences were compiled using BioEdit^6^ where they were cleaned to find the open reading frame and to keep exclusively the coding sequence (CDS) by removing the flanking untranslated regions (UTRs). Nucleotide sequences were aligned using the online version of MAFFT V7 (Katoh et al., 2019) with a gap open penalty of 3.0 and an offset value of 1.0. All other default settings were used without further modification. Maximum Likelihood (ML) phylogenetic analyses were done using IQ-Tree through the W-IQ-TREE portal^7^; Trifinopoulos et al., 2016). The molecular evolution model that best fit the data was calculated using the ModelFinder tool incorporated in IQ-TREE (Kalyaanamoorthy et al., 2017). The Ultrafast Bootstrap (UFBS) of 1000 pseudo-replicas also implemented in IQ-TREE was used to calculate branch support (Hoang et al., 2018). Phylogenetic trees obtained were visualized and edited on FigTree^8^ and labeled in Photoshop Illustrator CC 2019. Names of previously reported sequences were kept as published or with the original codes extracted from the databases. Gene names for the homologs isolated in this work were assigned based on their higher similarity to the *AtMYB114* canonical *Arabidopsis* gene, over the other three paralogs. In turn, they have all been named *MYB114-like*. Two analyses are presented, one with a comprehensive outgroup (SG4, SG5, SG7, and SG15) and all SG6 homologs including the monocot *R2R3-MYB* SG6 gene representatives (Supplementary Figure 1), and one restricted to SG6 homologs using *Arabidopsis thaliana MYB123* as outgroup (Figures 2, 3).

### Protein Sequence Analysis

**FIGURE 4:**
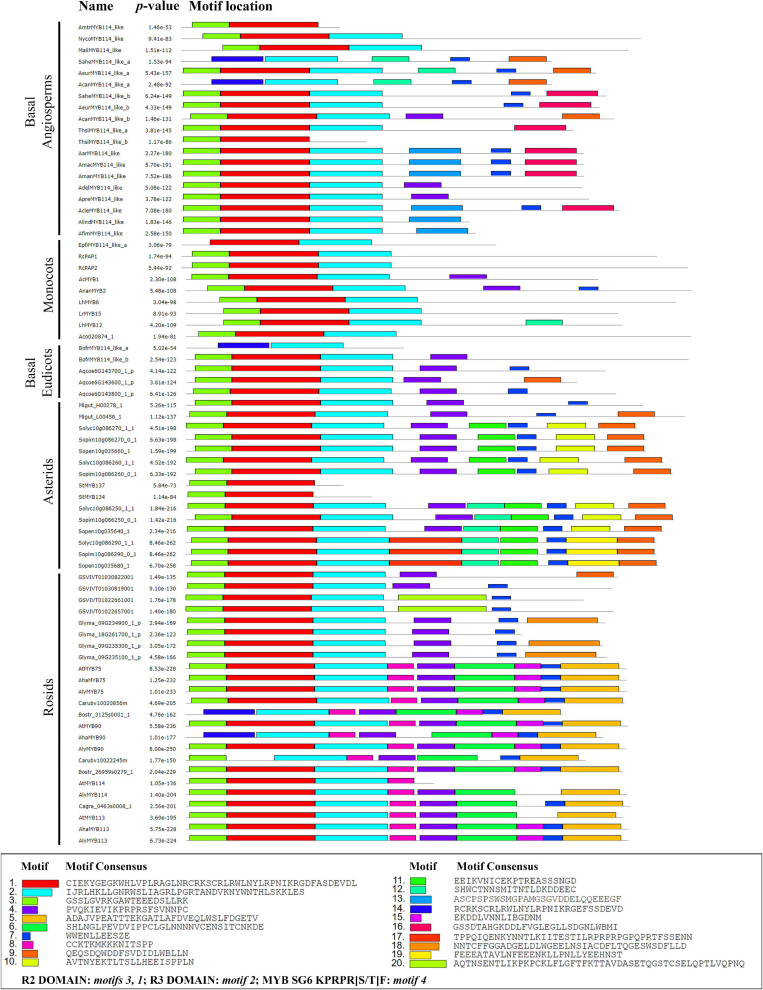
MEME analysis of selected SG6 R2R3-MYB proteins from core and non-core eudicots. Motifs found are indicated by color boxes. The sequence corresponds to the consensus, that is the amino acids occurring at higher rates in the motif. The R2 domain corresponds to motifs 1 and 3; R3 domain corresponds to motif 2. For gene codes, see Figures 2, 3 and Supplementary Table 1.

**FIGURE 5:**
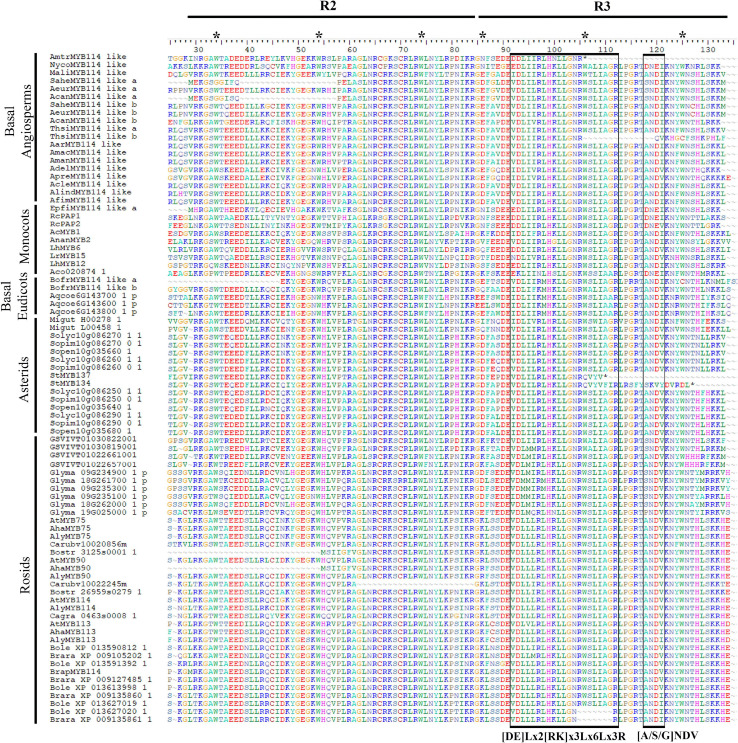
Protein sequence alignment of the R2 and R3 MYB domains from Subgroup 6 R2R3MYB proteins of selected basal angiosperms, monocots, basal eudicots and core eudicots. Asterisks represent conserved tryptophan residues. Left box indicates bHLH interacting motif. Right box indicates specific anthocyanin promoting MYB motif. For gene codes, see Figures 2, 3 and Supplementary Table 1.

In order to verify whether the previously reported domains for R2R3-MYB proteins were retained in all angiosperms and specifically if they were found in the Aristolochiaceae protein homologs, a MEME analysis was carried out. For this, the amino acid sequences were permanently translated using BioEdit. The resulting file was then introduced to the Multiple Em for Motif Elicitation (MEME) online server^9^ and analyzed using the default parameters and enquiring for the top 20 motifs. The motifs retrieved by MEME are reported according to their statistical significance. Within the given sequences, the MEME suite finds the most statistically significant (low *e*-value) motifs first. All SG6 *R2R3-MYB* sequences used in the phylogenetic analysis were included in this analysis (Figure 4). In addition, detailed amino acid alignments for the R2 and R3 domains as well as for the characteristic motif 4 of SG6 were exported from BioEdit (Figure 5 and Supplementary Figure 2).

### Expression Analyses by Reverse Transcriptase – PCR (RT-PCR)

Expression of SG6 *R2R3-MYB* homologs was assayed using RT-PCR on dissected perianth parts in different floral developmental stages from four selected species. Namely, *Saruma henryi*, *Asarum canadense* and two members of *Aristolochia* (*A. fimbriata* and *A. manshuriensis*). We chose them as they represent different floral groundplans and include contrasting patterns in the variation range for color display in the Aristolochiaceae, as will be described below.

*Saruma henryi* flowers were taken in early June 2019 from plants growing outdoors at the Botanischer Garten Dresden (Dresden, Germany), with approximately 12 h daylight and ca. 16°C; floral buds in pre-anthesis of ca. 0.8 cm were dissected, separating the bright yellow petals from the green sepals (Supplementary Figure 3A).

*Asarum canadense* flowers were collected from living collections at the Arnold Arboretum at Harvard University (Roslindale, MA, United States; Plant ID: 204-2006^∗^MASS-A). Sepals were taken at four different stages, as follows: stage S0, when the inner surface of the sepals was still yellow; S1, when purple color was first evident; pre-anthesis, when sepals were most intensely purple; and anthesis, when purple accumulation decreased (Supplementary Figure 3B).

*Aristolochia fimbriata* flowers were taken from plants cultivated under 16 h light and ca 22°C at the Evo-Devo lab (University of Antioquia, Medellín, Colombia). They were dissected in two developmental stages: S6 with completely green perianth and S9 (or pre-anthesis) flowers with pigmented flowers displaying intermixed yellow lines separating dark purple sections in the limb, a mostly purple tube, and a purple spotted predominantly pale-yellow utricle (Stages follow Pabón-Mora et al., 2015). The three perianth portions (utricle, tube and limb) were dissected accordingly (Supplementary Figure 3C).

*Aristolochia manshuriensis* flowers were collected in early May 2019 from living collections at the Arnold Arboretum at Harvard University (Roslindale, MA, United States; Plant ID: 424-87^∗^D), with approximately 10 h daylight and 10°C. Floral buds in two different developmental stages in pre-anthesis, in addition to flowers in anthesis were collected. The perianth at the S2 stage, with completely green/yellow perianth was used without further dissection between limb, tube and utricle, as no differences in color were evident. Conversely, the S9 perianth showed color difference in the inner surface of the utricle (dark purple), the tube (yellow and purple) and the limb (yellow); these three portions were dissected accordingly. Finally, only the yellow limb was dissected in anthetic flowers (Supplementary Figure 3D).

**FIGURE 6:**
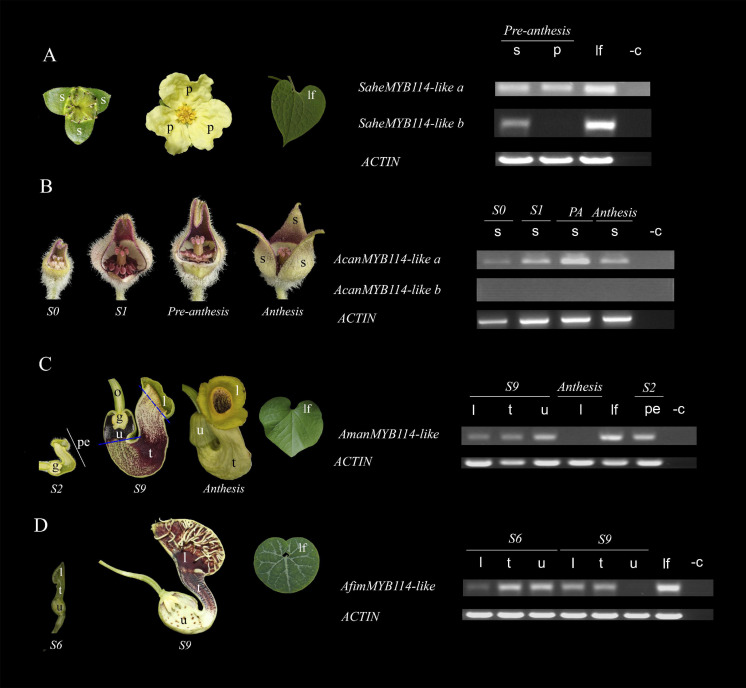
Expression of SG6 *R2R3-MYB* genes in **(A)** *Saruma henryi.* **(B)** *Asarum canadense*. **(C)** *Aristolochia manshuriensis*. **(D)** *A. fimbriata*. *ACTIN* was used as a positive control. *l*, limb; *lf*, leaf; *p*, petal; *pe*, perianth; *s*, sepal; *t*, tube; *u*, utricle; -c, amplification reaction without cDNA (negative control).

Total RNA from the individual dissected floral portions described above was extracted using TRIsure, following the manufacturer’s protocol (Bioline, London, United Kingdom). All RNA extractions were performed right after tissue collection in liquid nitrogen. RNA was quantified using a Nanodrop 2000 (Thermo Fisher Scientific). Total RNA obtained was treated with DNAseI (Roche, Switzerland) to remove genomic DNA contamination. cDNA was synthesized using the SuperScript III reverse transcriptase kit (Invitrogen, Carlsbad, CA, United States). One μg of RNA was used in every reaction carried, incorporating oligodT primers and following the manufacturer’s protocol. PCR was performed using 1 μl of the undiluted cDNA previously obtained and specific primers designed for the SG6 *R2R3-MYB* homolog genes identified in each species (Supplementary Table 2). Standardization of melting temperatures and cycles was done in order to determine the amplification reaction peak and saturation. For all species, the thermal cycling regime consisted of one initial step at 95°C for 5 min, 30 amplification cycles repeating the three step: 95°C for 30 s, Tm for 30 s, and 72°C for 50 s, and a final extension step at 72°C for 10 min. Melting temperatures (Tms) were as follows: for *Saruma henryi* PCRs, 48°C; for *Aristolochia fimbriata* and *Asarum canadense* PCRs, 54°C; and, for *A. manshuriensis* PCRs, 56°C. Experiments were carried in a MultiGene OptiMax thermocycler (Labnet International, Edison, NJ, United States). PCR products were run in 1.5% agarose gels with 1X TAE, stained with ethidium bromide. A *MyGel mini* (Accuris instruments, Edison, NJ, United States) electrophoresis camera was used, and results were visualized and digitally photographed using a Whatman Biometra BioDocAnalyzer (Göttingen, Germany). Original electrophoresis results are shown without brightness or contrast modifications (Figure 6). Quantitative data was obtained by Image J analyses of pixel intensity compared to *ACTIN* expression for all samples (Supplementary Figure 4). This was done following Wittall et al. (2006).

### Differential Gene Expression by RNA-Seq in *Aristolochia fimbriata*

*De novo* transcriptomes from *Aristolochia fimbriata* were generated as follows. Each transcriptome was obtained from three independent biological replicates of the dissected portions of the sepal-derived perianth, namely, the limb, the tube, and the utricle, at S6 and S9. The experiment was conducted in order to assess differentially expressed genes (DEGs) between the three portions of the perianth at two different developmental stages. Total RNA from the two dissected floral stages was extracted using TRIzol reagent (Invitrogen). The RNA-seq experiment was conducted using the Truseq stranded mRNA library construction kit (Illumina) and sequenced on a NovaSeq 6000 system reading 100 bp, paired-end reads. Read cleaning was performed with PRINSEQ-LITE (v0.20.4) available at http://printseq.sourceforge.net, with a quality threshold of Q30 at both ends and only keeping those longer that 70 bases after quality trimming. Contig assembly was computed using the Trinity package following default settings. Transcriptome assembly was performed for each perianth portion, in both developmental stages (Supplementary Table 3). In addition, a combined global transcriptome from all experiments was assembled as a reference with the following metrics: total assembled bases: 85.608.833 bp; total number of contigs (>101 bp): 118.941; average contig length: 719 bp; contig N50: 14.432 sequences ≧ 1.823 bp; contig N75: 31.828 sequences ≧ 746 bp; contig GC%: 42.71%.

**FIGURE 7:**
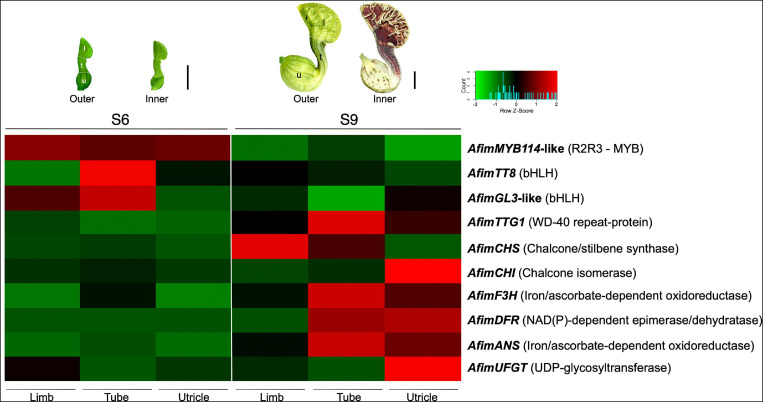
Expression analysis of genes involved in flavonoid production in *Aristolochia fimbriata*. The heatmap was generated based on normalized RNA-seq data. Ten genes were analyzed, six structural genes and four regulatory genes involved in the flavonoid biosynthetic pathway. Two different developmental stages (S6 and S9) and three portions of the perianth (limb, tube, and utricle) were compared. The color codes indicate upregulated (red) and downregulated (green).

To estimate the relative abundance of the assembled contigs, cleaned reads were mapped against the *de novo* assembled dataset implementing the algorithm Kallisto v.0.46.0 with default settings^10^. Kallisto quantifies transcript expression normalizing the relative abundance of each contig/transcript using the transcript per million (TPMs) metrics (Bray et al., 2016; Supplementary Table 4). Homologs for the bHLH and the WD40 families as well as for all enzymes were identified by reciprocal BLASTN searches. The homology for the transcription factors was confirmed by phylogenetic analyses (Supplementary Figures 5–7 and Supplementary Tables 5, 6). The relative abundance of *AfimMYB114*-like, *AfimTT8, AfimGL3-like*, *AfimTTG1, AfimCHS, AfimCHI, AfimF3H, AfimDFR, AfimANS*, and *AfimUFGT* transcripts was used to identify their expression level in each portion of the *Aristolochia fimbriata* perianth. This expression was calculated for the six generated transcriptomes corresponding to the utricle, the tube, and the limb at two different developmental stages (S6 and S9). Expression data from each sample was used to construct the heatmaps using the Shinyheatmap program (Figure 7)^11^ (Khomtchouk et al., 2017).

### Gene Annotation and Pathway Mapping

**FIGURE 8:**
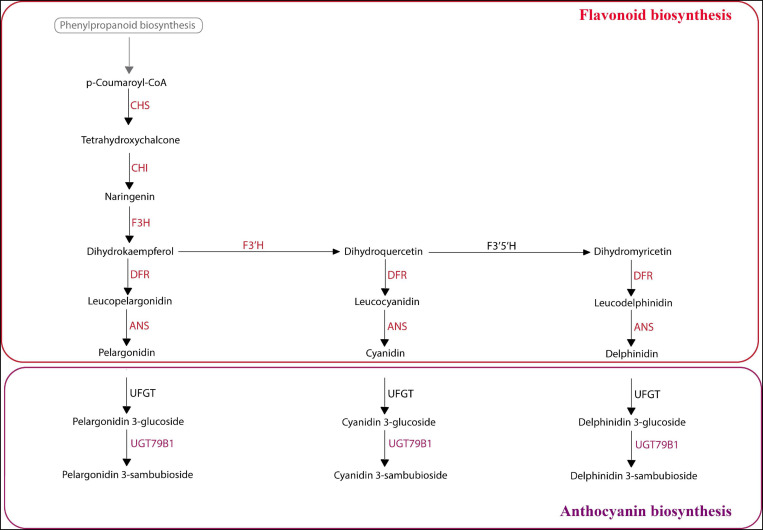
KEGG analysis of selected Aristolochiaceae species using predicted peptides. Summary flavonoid/anthocyanin biosynthetic pathway based on KEGG results. Colored enzyme names (in red and purple) represent those enzymes present in the peptide data from the mixed reference transcriptome in most analyzed species. For exceptions, see results. In black are the enzymes that were not found. ANS, Anthocyanidin synthase; CHI, Chalcone isomerase; CHS, Chalcone synthase; DFR, Dihydroflavonol 4-reductase; F3H, Flavanone-3-hydroxylase; F3′H, Flavonoid-3′-hydroxylase; F3′5′H, Flavonoid-3′5′-hydroxylase; UFGT, Anthocyanidin 3-O-glucosyltransferase; UGT79B1, Anthocyanidin 3-O-glucoside 2^″′^-O-xylosyltransferase.

Genes of the flavonoid and anthocyanin biosynthetic pathway were plotted using the KAAS tool of the KEGG database (Kanehisa and Goto, 2000). Previously obtained transcriptomes for different Aristolochiaceae species were translated to amino acid sequences and partitioned in several files due to size constrains. TransDecoder software^12^ was used for transcriptome translation. Protein sequences were submitted to the KEGG website^13^. The resulting analyses include the mapping of all enzymes found for the flavonoid and anthocyanin pathways for each species (Figure 8).

## Results

### Subgroup 6 *R2R3-MYB* Gene Evolution

First, we wanted to assess if the distantly related Aristolochiaceae had homologs from the SG6 *R2R3-MYB* genes reported in *Arabidopsis thaliana* (Brassicaceae) as they are central components of the MBW complex. The directed search using the SG6 *R2R3-MYB* canonical genes that integrates the MBW complex in *Arabidopsis thaliana* to assess Aristolochiaceae homologs resulted in 16 *R2R3-MYB* putative hits. In order to reconstruct the evolution of the SG6 *R2R3-MYB* genes and to verify homology, other genes from representative angiosperms were also retrieved from online databases and our own transcriptomes. Altogether, this sampling resulted in a total of 155 sequences. Namely, 3 gene sequences from early divergent angiosperms (ANA), 16 from Aristolochiaceae, 23 from monocots, 10 from basal eudicots and Chloranthaceae, and 103 sequences from core eudicots (Supplementary Figure 1 and Figures 2, 3). A first phylogenetic analysis with a comprehensive outgroup (SG4, SG5, SG7, and SG15) and all SG6 homologs including the monocot *R2R3-MYB* SG6 gene representatives (Supplementary Figure 1) allowed us to confirm SG6 homologs with a bootstrap support (BS = 90). A second phylogenetic analysis excluded the closely related subgroups to concentrate only in the SG6 genes. Following the first analysis *AtMYB123* was used as outgroup (Figures 2, 3). Newly identified genes were named as *MYB114-like*, as they resulted to be more similar in protein sequence to AtMYB114 than to any of the other three canonical *Arabidopsis* copies AtMYB75, AtMYB90 or AtMYB113. Our goal was to use a name that indicates their affinities to SG6 and the corresponding canonical *Arabidopsis* genes. All other genes in the tree retain the previously published names or codes.

The ML analysis of the SG6 *R2R3-MYB* genes is mostly consistent with the phylogenetic relationships of the major lineages sampled (Supplementary Figure 1 and Figures 2, 3). The exceptions are found in monocot homologs identified as SG6 homologs, several of which resulted as sister group to all other *R2R3-MYB SG6* genes (Figure 2 and Supplementary Figure 1). For the most part SG6 homologs reconstruct the evolution of angiosperms, having early diverging SG6 homologs as sister to Chloranthaceae and eudicot genes, and within eudicots representative subclades of asterid and rosid sequences (Figures 2, 3). Interestingly, the reconstruction of the SG6 *R2R3-MYB* gene evolution shows a larger number of local or species-specific duplications than large-scale duplications across angiosperms. In the Aristolochiaceae, the SG6 *R2R3-MYB* genes are mostly found as single copy genes and have undergone one duplication prior to the diversification of *Saruma* and *Asarum* resulting in the *MYB114-like a* and *MYB114-like b* clades (Bootstrap support BS = 84; Figure 2). However, one of the *A. canadense* copies (hereafter named *AcanMYB114-like*) comes out in an odd position as sister to all other Aristolochiaceae sequences, rather than nested in any of the two *MYB114-like* clades mentioned above. An additional species-specific duplication was found in *Thottea siliquosa* (BS = 100; Figure 2). The sequences from *T. siliquosa* are sister to those from species of *Aristolochia* subg. *Siphisia* and *Aristolochia* subg. *Aristolochia*, mirroring the phylogenetic sister-group relationships of these two genera. The sequences from species belonging to *Aristolochia* subg. *Pararistolochia* have a large number of non-synonymous changes and occupy an odd position as they cluster with one monocot sequence from *Allium cepa* (Figure 2). Nevertheless, in all analyses, they fall as members of the SG6 subgroup (Supplementary Figure 1 and Figure 2).

Additional large-scale duplications found in this gene linage occur in particular core eudicot groups. Some of them are linked to the diversification of the genus *Solanum* (Solanaceae), resulting in the *SolanumMYB114-like a* (BS = 100), *SolanumMYB114-like b* (BS = 100), *SolanumMYB114-like c* (BS = 100), and *SolanumMYB114-like d* (BS = 89) clades (Figure 3). Independent duplications have occurred in the Brassicales for Brassiceae (including *Brassica*) and Camelinae (including *Arabidopsis*) members (Figure 3). Three duplications have occurred in the Camelinae resulting in the *MYB75* (BS = 99), *MYB90* (BS = 71), *MYB113* (BS = 100), and *MYB114* (BS = 100) clades, which include the canonical *Arabidopsis* paralogs. There are two additional independent duplications in Brassiceae which we have labeled *Brassica MYB113/114-like a* (BS = 100), *MYB113/114-like b* (BS = 100) and *MYB113/114-like c* (BS = 100). Perhaps more inclusive sampling from more diverse species across the Brassicales will allow to assess the exact time point of these duplication events.

Additional species-specific duplications have occurred in *Aquilegia coerulea* (Ranunculaceae), *Bocconia frutescens* (Papaveraceae), *Carica papaya* (Caricaceae), *Daucus carota* (Apiaceae), *Glycine max* (Fabaceae), *Gossypium hirsutum* (Malvaceae), *Hedyosmum goudotianum* (Chloranthaceae), *Lilium ‘hybrid’* (Liliaceae), *Medicago truncatula* (Fabaceae), *Mimulus guttatus* (Phrymaceae), *Nelumbo nucifera* (Nelumbonaceae), *Petunia hybrida* (Solanaceae), *Populus trichocarpa* (Salicaceae), *Prunus persica* (Rosaceae), *Sarcandra chloranthoides* (Chloranthaceae), *Solanum tuberosum* (Solanaceae), *Streptosolen jamesonii* (Solanaceae), *Tropaeolum longifolium* (Brassicaceae), and *Vitis vinifera* (Vitaceae) (Figures 2, 3).

### Comparative Analysis of the Subgroup 6 R2R3-MYB Protein Sequences

Next, we wanted to verify if the typical R2 and R3 domains were present across all homologs and test for the presence of other conserved motifs that could point to conserved functions in distantly related plant lineages. The *R2R3-MYB* genes present a highly conserved N-terminal R2R3 domain that binds DNA. We found that the R2 domain is recovered in the motifs 1 and 3, while the R3 domain is recovered in motif 2 in our analysis (Figures 4, 5). This was verified in the ScanProsite database^14^ where motifs 1, 2 and 3 correspond to DNA binding HTH MYB-type domain. Interestingly, motifs 1, 2 and 3 comprising the R2R3 domains are present in most homologs analyzed (Figure 5): The exceptions are motif 1 and 3, lacking in the *Saruma henryi* SaheMYB114-like a, the *Asarum canadense* AcanMYB114-like a and the *Bocconia frutescens* BofrMYB114-like a. On the other hand, motif 2 is lacking in the *Thottea siliquosa* ThsiMYB114-b and the *Amborella trichopoda* AmtrMYB114-like homolog as well as in both *Solanum tuberosum* homologs StMYB134 and StMYB137 (Figure 4). These cases likely represent variants with deletions complicating motif recovery by MEME, because visual inspection in the amino acid alignments do show partial regions retained (Figure 5).

Motif 4 (KPRPRS/TF) which has been regarded as predictive of SG6 homologs is in fact present in most eudicot sequences, but in early diverging angiosperms can only be found intact in AdelMYB114-like, ApraMYB114-like and AcanMYB114-b, while there are several non-synonymous substitutions in the rest of the taxa sampled (KLPNSV; Supplementary Figure 3). Monocot homologs also lack an intact SG6 predictive motif and present a divergent RPQPR/K. Other motifs identified by MEME seem to be characteristic of some groups. For instance, motifs 5, 6, 8, and 15 can help recognize R2R3-MYB proteins from Brassicales (Figure 4). Motifs 9, 10, and 19 are characteristic of several proteins in the Solanales. Finally, some motifs seem to be species-specific, as motif 18 appears only in *Glycine max* homologs and motif 20 is only present in *Vitis vinifera* proteins. None of the new motifs identified has any reference functions; however, these conform shared protein sequences that will have to be tested in the future for specific roles.

### Expression Analysis of the Subgroup 6 *R2R3-MYB* Genes in Selected Aristolochiaceae

The next step in our study was to analyze the expression patterns of the SG6 *R2R3-MYB-like* genes in different Aristolochiaceae species. For this, the following four Aristolochiaceae species were selected based on the color variation of their perianth: *Saruma henryi*, with a biseriate perianth formed by three green sepals and three yellow petals; *Asarum canadense*, with a uniseriate perianth formed by three dark purple fused sepals; *Aristolochia fimbriata*, with a uniseriate perianth formed by three sepals with an inner surface mainly purple and yellow; and *A. manshuriensis* with a uniseriate perianth formed by three sepals with an inner surface with mainly green, dark purple and yellow tones (Figure 6). Whenever possible, different developmental stages and perianth parts were sampled (see section “Materials and Methods”). *ACTIN* was used as a positive control in all samples.

In *Saruma henryi*, the SG6 *R2R3-MYB-like* gene *SaheMYB114-like-a* is broadly expressed in sepals, petals and leaves, but *SaheMYB114-like-b* is restricted to the green sepals and the leaves. No expression of this homolog was detected in the yellow petals. In *A. canadense*, *AcanMYB114-like-a* is found in sepals through all developmental stages, with a peak of gene expression, which coincides, with a visual peak of anthocyanin accumulation in the perianth during pre-anthesis. No expression was found for *AcanMYB114-like-b*. In *A. manshuriensis*, *AmanMYB114-like* is expressed early in the light green perianth prior to any purple accumulation in its inner surface at S2. The same broad expression is found for *AmanMYB114-like* at S9 in the green limb, the reddish tube, and especially in the dark purple utricle. Expression of *AmanMYB114-like* is lacking in the yellow limb at anthesis. Importantly, *AmanMYB114-like* is actively expressed in the green leaves. Finally, in *A. fimbriata*, *AfimMYB114-like* is present in the limb, tube, and utricle at S6, by the time the inner perianth surface is still green throughout. Later, in flowers at S9, the expression of *AfimMYB114-like* becomes restricted only to the purplish limb and tube. Like its homolog, *AfimMYB114-like* is expressed in the green leaves (Figure 6).

### Differential Expression Analysis in the *Aristolochia fimbriata* Perianth

Homologs of all members forming the MBW complex were identified in the Aristolochiaceae (Supplementary Figures 5–7). Comparative expression levels of the regulatory (*AfimMYB114-like, AfimTT8, AfimGL3*, and *AfimTTG1*) and structural genes (*AfimCHS, AfimCHI, AfimDFR, AfimF3H, AfimANS*, and *AfimUFGT*) were tested in the limb, the tube, and the utricle at S6 and S9 floral developmental stages (Figure 7). Perianth at S6 flowers is completely green. In contrast, S9 flowers display differential color patterns in the perianth. The limb’s background is dark purple with yellow longitudinal and transversal stripes, the tube has dark purple and light yellow longitudinal stripes, and the utricle is mostly light yellow with a few dark purple spots scattered through it.

Most of the regulatory genes (*AfimMYB114-like*, *AfimTT8*, and *AfimGL3*) begin their expression even before the acquisition of the purple color in the perianth at S6. Importantly, their highest accumulation coincides with the early onset of purple coloration in the tube. At S6, none of the structural genes (i.e., *AfimCHS, AfimCHI, AfimDFR, AfimF3H, AfimANS*, and *AfimUFGT*) seem to be particularly active. However, most of these genes increase in expression at S9 concomitant with the reduced levels of the regulatory genes. Among structural genes, only *AfimCHS* seems to be highly expressed in the limb, while all others concentrate in the tube or the utricle (Figure 7). Interestingly, *AfimTTG1* was the only transcription factor of the putative MBW complex found to be highly expressed at the S9 developmental stage.

### Comparative Mapping of the Anthocyanin Biosynthetic Pathway Across Aristolochiaceae

Finally, we wanted to map our reference transcriptomes generated from mixed leaves, flowers and fruits to the flavonoid and anthocyanin pathways from the KEGG database, in order to see which transcription factors were present in selected Aristolochiaceae. First, the flavonoid pathway was analyzed. For *Saruma henryi*, *Asarum canadense, Asarum europaeum*, *Thottea siliquosa*, *Aristolochia deltantha*, *A. lindneri*, *A. macrophylla*, *A. manshuriensis*, and *A. praevenosa*, all the core flavonoid biosynthesis enzymes were present, except for the flavonoid 3’,5’-hydroxylase (CYP75A) which did not have a match in any of the peptides provided for any species (Figure 8). We noticed two additional enzymes lacking specific matches in two *Aristolochia* species, namely, the bifunctional dihydroflavonol 4-reductase/flavanone 4-reductase (DFR) and the anthocyanidin synthase (ANS) enzymes were missing from the *A. arborea* peptide dataset, and the same anthocyanidin synthase (ANS) could not be found in the *A. clematitis* dataset.

Next, the anthocyanin biosynthetic pathway was evaluated. A single enzyme of this pathway, the 3-O-glucoside 2*^″′^*-O-xylosyltransferase (UGT79B1) enzyme is present in all Aristolochiaceae evaluated, except in *A. arborea* (Figure 8). One interesting case is the UFGT enzyme, which performs flavonoid glycosylation and thus is critical for final anthocyanin production. This enzyme did not find predicted peptide matches in any of the species evaluated. However, a directed search using the RNA-seq data from *A. fimbriata* provided a match for this enzyme. In turn, predicted peptides may be less reliable than BLASTN searches for automatizing pathway predictions.

## Discussion

### *R2R3-MYB* Gene Diversification Is Likely Driven by Tandem Duplication Events in Core Eudicots

In *Arabidopsis thaliana*, four *R2R3-MYB* genes involved in the anthocyanin biosynthesis pathway have been reported, namely, *AtMYB75*, *AtMYB90*, *AtMYB113*, and *AtMYB114* (Stracke et al., 2001). All SG6 *R2R3-MYB* genes control early anthocyanin biosynthesis in vegetative tissues (Dubos et al., 2010) and overexpression of *MYB75* and *MYB90* by activation tagging can result in darker leaves and purple petals in *Arabidopsis* (Borevitz et al., 2000). Similarly, overexpression of the *Eutrema salsugineum EsMYB90* gene in tobacco and *Arabidopsis* results in anthocyanin accumulation and upregulation of structural genes of the flavonoid biosynthesis pathway (Qi et al., 2020). Other studies in red-skinned pear (*Pyrus bretschneideri*) demonstrated that an *R2R3-MYB* homolog, *PyMYB114*, is responsible for anthocyanin biosynthesis in the red fruit skin (Yao et al., 2017). Similarly, the overexpression of *AtMYB75* induces anthocyanin production in *Solanum lycopersicum* starting in the seedling until the development of stems and leaves (Zuluaga et al., 2008). Likewise, an *AtMYB113* homolog in *Solanum melongena, SmMYB113*, induces anthocyanin accumulation by binding to the promoter of the chalcone isomerase (SmCHI) and dihydroflavonol 4-reductase (SmDFR) enzymes (Li et al., 2017). Outside eudicots the SG6 *R2R3-MYB* homologs have been less studied. The *RcPAP1* and *RcPAP2 Cattleya* hybrids homologs do activate the anthocyanin synthesis pathway in the flowers (Li et al., 2020). Altogether, the present data on gene homologs belonging to the SG6 *R2R3-MYB* genes suggest that they perform key early roles in the control of the anthocyanin pathway in different angiosperms and in different plant organs. However, to date no comprehensive phylogenetic framework including the functionally analyzed *SG6 R2R3-MYB* core eudicot homologs is available, and it is less clear if their roles outside of eudicots and monocots are maintained.

Our analysis shows that many model core-eudicots including members of the Solanaceae and Brassicaceae have undergone local duplication events (Figure 3). For instance, we identified three duplications exclusive to *Solanum* yielding four (a–d) *SolanumMYB114-like* clades (Figure 3). Interestingly, *Solanum tuberosum*, a well-known polyploid, has only two gene copies, compared to the diploid *S. lycopersicum*, which has four copies, one on each of the *SolanumMYB114-like* clades. This reinforces the hypothesis that the evolution of *Solanum MYB* genes has been driven by tandem duplications and not by whole-genome duplication events (Sun et al., 2019).

Similar local duplications were identified in Brassicales. Three duplications resulted in the diversification of the *MYB90*, *MYB75* and *MYB113*, and *MYB114* clades in *Arabidopsis* and close relatives belonging to the Camelinae (Guo et al., 2017). Independent additional Brassiceae specific duplications resulted in three major clades (*MYB113*/*MYB114 a, b*, and *c*) and additional species-specific duplications in *Brassica* species are recorded. In turn, our results show that *B. oleracea* and *B. rapa* have more gene copies than *Arabidopsis*. This condition could be related to independent genome duplication events associated with the two genera (Wang et al., 2015).

Local duplications are extremely common in other basal and core eudicots evaluated. We found that 17 of the total species evaluated presented species-specific duplications. Such tandem duplications had also been identified in *Aquilegia coerulea*, *Medicago truncatula*, *Populus trichocarpa*, and *Vitis vinifera*, suggesting that small-scale duplications have been important for the expansion of this gene subfamily (Wilkins et al., 2009; Du et al., 2015).

Conversely, the SG6 *R2R3-MYB* genes in monocots and early divergent angiosperms show few to none local duplications. We speculate that tandem duplications are less frequent or that strong selection can be acting upon gene copies, limiting the occurrence or the retention of duplicates. Our ML analysis points to a single duplication in the Aristolochiaceae that predates the *Asarum/Saruma* diversification, which results in the *MYB114-like a* and *b* clades, and one species-specific duplication in *Thottea siliquosa* (Figure 2). All remaining Aristolochiaceae and other magnoliids sampled have single copy SG6 *R2R3-MYB* genes. Gene duplications predating the *Asarum/Saruma* diversification have also been detected in the *class II TCP CIN2* genes, involved in the regulation of cell division and floral patterning (Pabón-Mora et al., 2020). It has been previously reported that *Asarum* and *Saruma*, the two members of the subfamily Asaroideae, have a genome size about ten times larger than in species of *Aristolochia* (Bliss et al., 2013). This suggests that gene copy number could be related to larger genome sizes due to whole-genome duplication (WGD) events. Less is known about the genome size of *Thottea siliquosa*, but *class II TCP CIN3* genes are also duplicated in this species, perhaps pointing to unexplored tandem repeats or WGD events occurring in this taxon (Pabón-Mora et al., 2020).

### Unusual Domains May Explain Divergent Phylogenetic *R2R3-MYB* Gene Placement

Our analysis of the R2 and R3 domains found in the N-terminal portion of the MYB-R2R3 proteins focused on the three spaced tryptophan residues that form a hydrophobic cluster critical for their function. These tryptophan residues were observed in all the sequences analyzed in this work. As reported by Du et al. (2015), the first residue of the R3 domain was seen to be either phenylalanine (F) or isoleucine (I). The latter two helices of each domain form the HTH structure (helix-turn-helix) and the third is important in the DNA interactions (Wang et al., 2015). The R2 and R3 domains, as reported by Stracke et al. (2001), correspond to motifs 1, 2, and 3 found in our MEME analysis. In turn, it is not surprising that these highly conserved domains with DNA binding function are found in most homologs (Figure 4). For the eudicot anthocyanin-promoting MYBs, Liu et al. (2016) report the motif [A/S/G]NDV located inside the R3 domain. This work also reports the bHLH interacting motif in the conserved region of the R3 domain ([DE]Lx2[RK]x3Lx6Lx3R) (Figure 5).

Some authors have reported specific motifs for the SG6 R2R3-MYB proteins. Stracke et al. (2001) reported the motif KPRPR[S/T]F at the C terminal portion. This domain is located inside motif 4 from the MEME analysis, and it is present almost intact in eudicots but has dramatic variation in monocots (RPQPR) and basal angiosperms (KPNLC; Supplementary Figure 3). Importantly, in the Aristolochiaceae, motif 4 was only found intact in AcanMYB114-like b (KPQPRT), ApraMYB114-like and AdelMYB114-like (KPKPRAL) proteins. This is particularly interesting since all three homologs do not cluster with the other Aristolochiaceae homologs in our phylogenetic hypothesis, suggesting that the retention of motif 4 is rare in Aristolochiaceae. Conversely, motif 7 seems to be more deeply conserved across SG6 R2R3-MYB proteins and can serve hereafter to identify members of this group more easily in non-model species (Figure 4). Available functional data does suggest, however, that the canonical SG6 motif KPRPR[S/T]F from eudicots may not be indispensable for the role of these transcription factors in anthocyanin synthesis. At least in monocots the contribution of SG6 R2R3 MYB proteins to purple color in the lip occurs independently of the modifications in the motif (RPMVIR in RcPAP1 and RTKAIR in RcPAP2; Supplementary Figure 2; Li et al., 2020).

### Expression of *R2R3 MYB* Genes Is Correlated With Anthocyanin Accumulation and Floral Color Patterning

Anthocyanins are best known for conferring different colors in plants, such as red, purple, and blue. Flavonoid biosynthesis enzymes are typically distinguished into two groups: the early biosynthesis genes (EBGs) and the late biosynthesis genes (LBGs) (Fang et al., 2019). *R2R3-MYB* proteins can directly regulate early stage enzymes (SG7 *R2R3-MYBs*), whereas MBW complexes activate later biosynthetic steps (Dubos et al., 2008). These complexes include a R2R3-MYB protein (usually a SG6 protein), a bHLH protein and a WD40 protein, which is not catalytic (Li, 2014). In *Arabidopsis*, the WD40 TF homolog is AtTTG1. The bHLH TFs include AtTT8, AtGL3, and AtEGL1, which have partially redundant functions. All SG6 *R2R3-MYB* factors, namely AtMYB75, AtMYB90, AtMYB113, and AtMYB114 participate in the MBW complex for anthocyanin pathway activation in *Arabidopsis.* Their ectopic expression increases anthocyanin production when associated with bHLH and AtTTG1 (WD40) TFs (Petroni and Tonelli, 2011). Here we wanted to evaluate if the homologs in early divergent angiosperms play similar roles in floral pigmentation.

We found two SG6 *R2R3MYB-like* genes copies for *Saruma henryi* and *Asarum canadense*, and one copy for *Aristolochia manshuriensis* and *A. fimbriata*. Our results on the expression of these genes showed that the SG6 *R2R3-MYB* homologs are expressed in early developmental stages in the sepals, independently of whether they are protective and green at maturity like in *Saruma* or acquire purple color and attract pollinators like in *Asarum* or *Aristolochia*. Such expression may be related to incipient anthocyanin accumulation for protection against UV radiation and cold temperatures (Sarma and Sharma, 1999; Petroni and Tonelli, 2011). This expression of SG6 *R2R3-MYB* genes in young green floral organs and immature fruits is relatively common. In kiwi (*Actinidia chinensis*) expression of *R2R3-MYB* genes has been observed in green fruits 7 days after anthesis, prior to any notable anthocyanin accumulation (Li et al., 2015). Yellow or white floral parts of *Aristolochia* flowers lack SG6 *R2R3-MYB* gene expression, consistent with low anthocyanin levels (Petroni and Tonelli, 2011). Conversely, dark purple or red perianth organs show high expression levels for these anthocyanin-related *R2R3-MYB* genes. These results indicate that the participation of SG6 *R2R3-MYB* transcription factors as part of the anthocyanin pathway is already in place in Aristolochiaceae. Finally, the differences in expression patterns between the close recent *R2R3-MYB* paralogs in *Saruma* and *Asarum*, suggests that duplications may have resulted in divergent expression of one of the duplicates. At least in *Asarum* one copy *AcanMYB114-like a* retains the role in anthocyanin production, while *AcanMYB114like b* is no longer expressed in the *Asarum* sepals suggesting functional differences after the duplication (Figure 6). On the other hand, *SaheMYB114-like a* is expressed in both the green sepals and the yellow petals. This suggests that SG6 *R2R3-MYB* transcription factors in the Aristolochiaceae are more pleiotropic than their equivalents in rosids and asterids.

Mixed purple/red and yellow perianths are relatively common in *Aristolochia*. It has been reported that anthocyanins and carotenoids can occur simultaneously in perianth organs. For example, in some Chilean *Mimulus* species, carotenoids produce the yellow background, while anthocyanins confer a darker coloration in the dorsal petal surface and some spotted areas in the flower throat and central petal (Davies et al., 2012). Mixed yellow and purple perianth in some Aristolochiaceae species may be the result of parallel carotenoid and anthocyanin production in specific floral domains. Thus, addressing how these genes acquire restricted spatio-temporal expression and what other factors can act as negative regulators, is critical to fully understand color patterning in Aristolochiaceae flowers.

Our comparative targeted transcriptomic analysis in two different developmental perianth stages from *A. fimbriata* evaluated four regulatory genes (*AfimMYB114-like*, *AfimTT8*, *AfimGL3*, and *AfimTTG1*) and six structural genes from the flavonoid biosynthetic pathway (*AfimCHS*, *AfimCHI*, *AfimF3H*, *AfimDFR, AfimANS*, and *AfimUFGT*). Phylogenetic analyses confirmed homology for all transcription factors (Supplementary Figures 5–7). The RNA-seq expression data confirmed that MYB and bHLH regulatory genes (*AfimMYB114-like*, *AfimTT8*, and *AfimGL3*) are up-regulated in the early developmental stages (S6), while *AfimTTG1* and the structural genes are up-regulated in the later stages of development (S9) (Figure 7). Both, the RT-PCR and the RNA-seq data point to upregulation of this pathway at S6 prior to any evident purple color accumulation. Importantly, the SG6 *R2R3-MYB* homolog, *AfimMYB114-like* is the only regulatory gene that is up-regulated in all three perianth parts (limb, tube, and utricle) in the S6 developmental stage. This indicates that the SG6 *R2R3-MYB* genes are good candidate transcription factors to activate early enzymes in the pathway, similar to the SG7 *R2R3-MYB* enzymes in *Arabidopsis*, and later as part of the MBW complex (Zhao et al., 2013). The bHLH homologs, *AfimTT8* and *AfimGL3-like* genes are up-regulated in the tube at S6, which is also the first part of the perianth to turn dark purple during development. Conversely, the WD40 homolog, *AfimTTG1* gene was found to be up-regulated in S9, but not S6. The correlated expression of the SG6 *R2R3-MYB* genes and the bHLH TFs in the absence of a WD40 partner suggests a simpler MB complex in early divergent species, when compared to a complete MBW complex in model core eudicots. Alternatively, a complex may not be needed until late (S9) developmental stages were anthocyanins are more actively produced. All of these are testable hypotheses for future functional analyses.

The WD40 transcription factor *TTG1* and all enzymes of the flavonoid pathway evaluated here are up-regulated in the S9 stage. *AfimCHS*, the critical starting factor of the flavonoid pathway, appears to be up-regulated in the tube and the limb at S9 flowers. All enzymes, except *AfimCHS*, are up-regulated in the utricle. Such contrasting level of regulation could be correlated to the predominantly yellowish background of the utricle, and the restriction of dark purple to brown color to small spots associated with nectar production.

### All Major Flavonoid and Anthocyanin Pathway Enzymes Are Present in Most Aristolochiaceae Species

The glycosylated forms of anthocyanidins, namely cyanidin, delphinidin, and pelargonidin are the most common anthocyanidins in flowering plants. Here, the absence/presence of the biosynthetic pathway enzymes for the formation of these three anthocyanins was evaluated for selected Aristolochiaceae species. Our *In silico* approach consisted on using predicted peptides from mixed transcriptomes resulting from total RNA extracted from leaves, flowers and fruits (if available) and mapping the complete routes in KEGG. These analyses can serve as a proxy to get preliminary observations on the conservation of complete pathways in non-model species. However, as the data comes from transcriptomic raw data, these are putative enzymes active at particular plant organs in specific developmental stages (see materials and methods).

Our results show that the enzyme Flavonoid 3′5′-hydroxylase is lacking in all examined species, which suggests that delphinidin is not produced. Delphinidin is better known for producing blue hues. For example, in *Hydrangea* flowers delphinidin-3-glucoside can form blue complexes by interacting with metal ions, especially Al^3+^ and Fe^3+^ (Yoshida et al., 2003; Schreiber et al., 2010), and blue is not normally part of the floral display in Aristolochiaceae.

All other major flavonoid pathway enzymes were found in most examined species, except in *Aristolochia arborea* and *A. clematitis*. KEGG results show that cyanidin and pelargonidin, two anthocyanidins mostly responsible for red and red-like colors (Chung et al., 2016), are synthesized in all species studied, except in *A. arborea* and *A. clematitis*. Red and dark red colors are common in the perianth of many *Aristolochia* species; nevertheless, there was no peptide predicted that matched an anthocyanidin synthase (ANS) in *A. clematitis* or in *A. arborea.* In addition, no match was found for the bifunctional enzyme Dihydroflavonol 4-reductase/flavanone 4-reductase (DFR) in *A. arborea.* Given that the plant tissue used for the transcriptomes contained a mix of flowers and leaves in all the species, it makes sense that we were able to map all major enzymes in the yellow-flowered *A. clematitis*, as these enzymes are present and active in leaves even if anthocyanins are not being produced in the flowers. However, the absence of the ANS and DFR in *A. arborea* and ANS in *A. clematitis* from the predicted peptides may reflect either a biological process, such as the impact of environmental factors to which plants were exposed when tissue samples were taken, or an artifact resulting from low match in peptide sequence prediction. To support the former scenario, it is important to note that anthocyanin synthesis in fruits is affected by environmental conditions, such as light, temperature and pH (Jaakola, 2013). Cauliflory in *A. arborea* results in the production of flowers at the ground level of shaded tropical forests; it has been reported that flavonoid/anthocyanin pathway structural gene expression (like CHS and F3H) can be lowered by shading (He et al., 2010). To support the latter assumption, it is important to highlight that the UFGT enzyme was not found in any of the evaluated species by KEGG. This may be due to high divergence between the predicted peptide for these non-model Aristolochiaceae species when compared to the references in the KEGG database. We were able to isolate this enzyme from directed searches in our own *A. fimbriata* RNA-seq data, suggesting that it is an artifact of the *In silico* translation for some proteins.

## Conclusion

Taken together, the phylogenetic reconstruction of the evolution of *R2R3-MYB* genes combined with the targeted expression analyses and the mapping of biosynthetic routes *in silico* shows that the flavonoid and early enzymes of the anthocyanin biosynthetic pathways are present and active in the Aristolochiaceae family. Importantly, single copy upstream regulators likely perform the same roles as those found duplicated in core eudicots. Moreover, when duplications were found, like in *Asarum* and *Saruma*, divergent expression of the copies is detected suggesting functional diversification after duplication. The fact that SG6 *R2R3-MYB* genes are found in leaves and sometimes even in yellow floral parts (like is the case for *SaheMYB114-like_a*) suggests more pleiotropic roles for these homologs when compared to their equivalents in rosids and asterids. Our results show that the SG6 *R2R3-MYB* single copy genes controlling the flavonoid biosynthetic pathways are simplified in the Aristolochiaceae and that a more complex regulation has evolved later in core eudicots with a larger copy number, such as in the Brassicales. Our data also shows that other members of the MBW complex are present as single copy genes and are active in Aristolochiaceae. The scenario that we present for SG6 *R2R3-MYB* genes poses the Aristolochiaceae as a suitable family to study complex color floral patterns with a relatively simple genetic bases with single copy regulators and a complete active pathway.

## Data Availability Statement

The original contributions presented in the study are publicly available. The datasets generated for this study can be found in the NCBI GenBank accessions MW125647-MW125662.

## Author Contributions

SM-G, HS-B, FG, and NP-M planned and designed the research, conducted fieldwork, and performed the experiments. SM-G, HS-B, NP-M, and JA executed the bioinformatics analysis. SM-G, HS-B, JA, FG, and NP-M analyzed the data, and wrote and approved the final manuscript. All authors contributed to the article and approved the submitted version.

## Conflict of Interest

The authors declare that the research was conducted in the absence of any commercial or financial relationships that could be construed as a potential conflict of interest.

## Footnotes

1 https://www.arabidopsis.org/

2 https://www.ncbi.nlm.nih.gov/

3 https://phytozome.jgi.doe.gov/pz/portal.html

4 https://sites.google.com/a/ualberta.ca/onekp/

5 http://planttfdb.gao-lab.org/

6 https://bioedit.software.informer.com/7.2/

7 http://iqtree.cibiv.univie.ac.at/

8 http://tree.bio.ed.ac.uk/software/figtree/

9 http://meme-suite.org/

10 https://pachterlab.github.io/kallisto/

11 https://github.com/Bohdan-Khomtchouk/shinyheatmap

12 https://github.com/TransDecoder/TransDecoder/wiki

13 https://www.genome.jp/kegg/

14 https://prosite.expasy.org/scanprosite/

## Supplementary Material

Supplementary Figure 1 Comprehensive Maximum likelihood analysis of the SG6 *R2R3-MYB* genes with an expanded outgroup including SG4, 5, 7, and 15. Outgroup used here is *AtMYB3*.

Supplementary Figure 2 Protein sequence alignment of SG6 R2R3-MYB selected proteins showing the diagnostic *R2R3-MYB* motif for subgroup 6 as reported by Stracke et al., 2001 boxed.

Supplementary Figure 3 Developmental stages sampled for RT-PCR of **(A)** *Saruma henryi*, **(B)** *Asarum canadense*, **(C)** *Aristolochia fimbriata*, **(D)** *A. manshuriensis. l*, limb; *p*, petal; *s*, sepal; *t*, tube; *u*, utricle.

Supplementary Figure 4 Image J quantitative expression analysis based on the original electrophoresis images obtained by RT-PCR with an unprocessed background.

Supplementary Figure 5 Maximum likelihood analysis of the *EGL1/GL3* bHLH genes across angiosperms. Yellow stars indicate large-scale duplication events. Color clades follow the conventions in the top left. The *Aristolochia fimbriata* homolog is pointed with a red arrow.

Supplementary Figure 6 Maximum likelihood analysis of the *TT8* bHLH genes across angiosperms. Color clades follow the conventions in the top left. The *Aristolochia fimbriata* homolog is pointed with a red arrow.

Supplementary Figure 7 Maximum likelihood analysis of the *TTG1* WD40 genes across angiosperms. Color clades follow the conventions in the top left. The *Aristolochia fimbriata* homolog is pointed with a red arrow.

Supplementary Table 1 Names, abbreviations, codes, and original databases for all sequences included in the SG6 R2R3 MYB phylogenetic analyses.

Supplementary Table 2 Name, sequence, and melting temperature of the primers used during RT-PCR analyses.

Supplementary Table 3 *De novo* assembly of the floral transcriptomes of *Aristolochia fimbriata* at two different developmental stages.

Supplementary Table 4 Expression of the candidate genes included in this study. Values correspond to the TPMs.

Supplementary Table 5 Names, abbreviations, codes, and original databases for all sequences included in the bHLH phylogenetic analyses.

Supplementary Table 6 Names, abbreviations, codes, and original databases for all sequences included in the WD40 phylogenetic analyses.
