# Supplementary figures and images for: Evolution of the Subgroup 6 R2R3-MYB Genes and Their Contribution to Floral Color in the Perianth-Bearing Piperales
Source: Front Plant Sci. 2021 Apr 9;12:633227. doi: 10.3389/fpls.2021.633227 (PMC8063865; doi:10.3389/fpls.2021.633227)

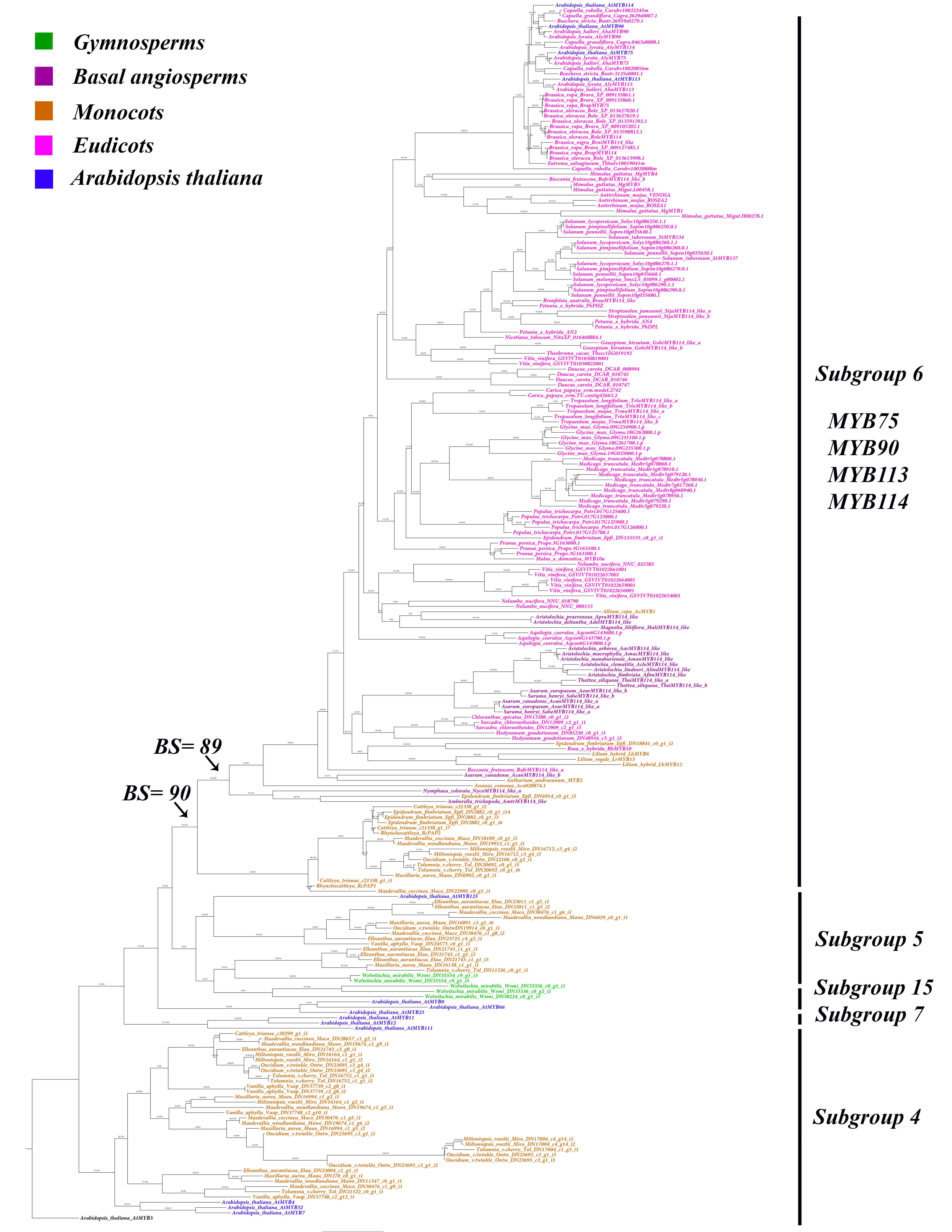

Supplement: Supplementary Figure 1 — Comprehensive Maximum likelihood analysis of the SG6 R2R3-MYB genes with an expanded outgroup including SG4, 5, 7, and 15. Outgroup used here is AtMYB3. [file Image_1.TIF]

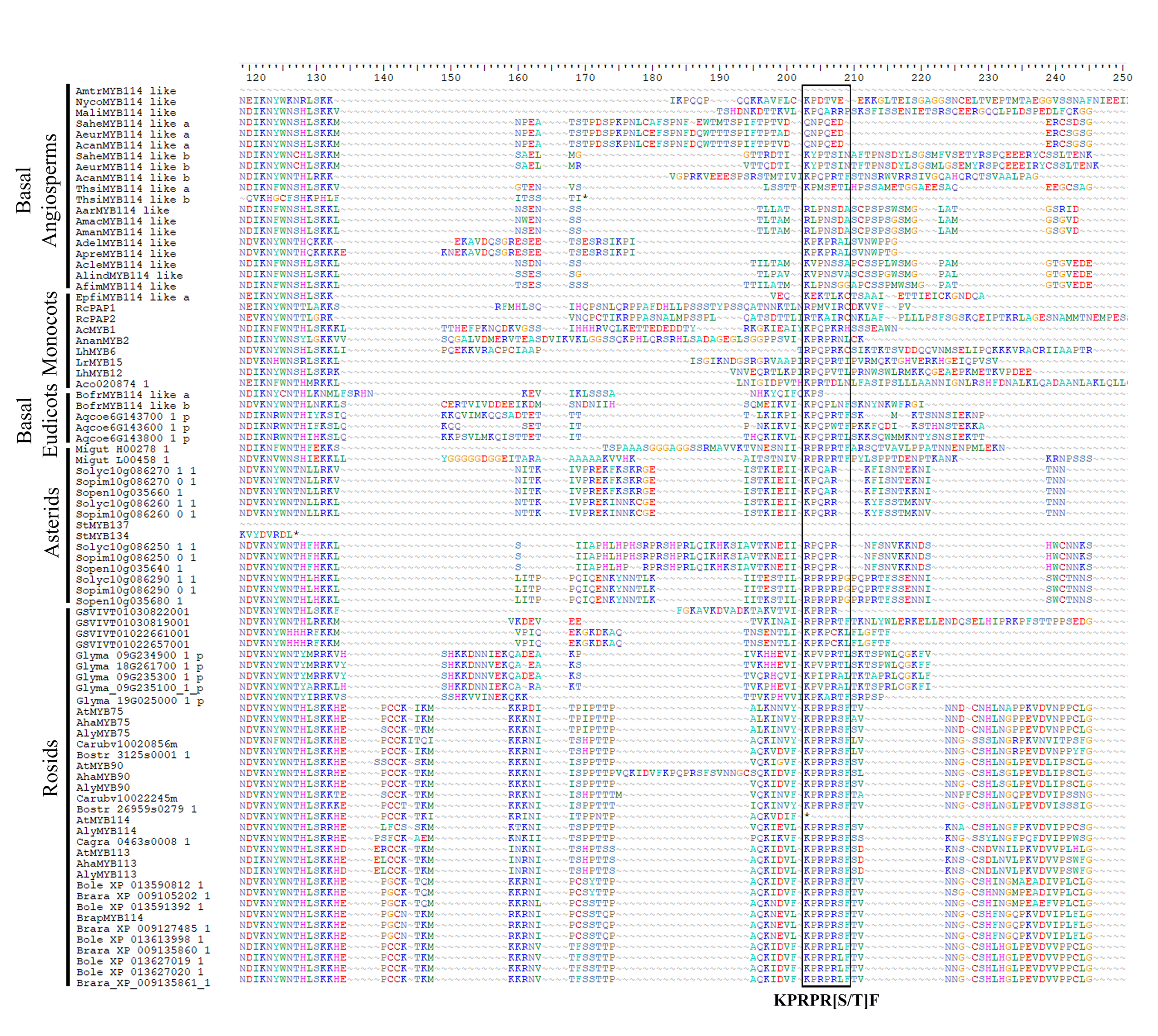

Supplement: Supplementary Figure 2 — Protein sequence alignment of SG6 R2R3-MYB selected proteins showing the diagnostic R2R3-MYB motif for subgroup 6 as reported by Stracke et al., 2001 boxed. [file Image_2.TIF]

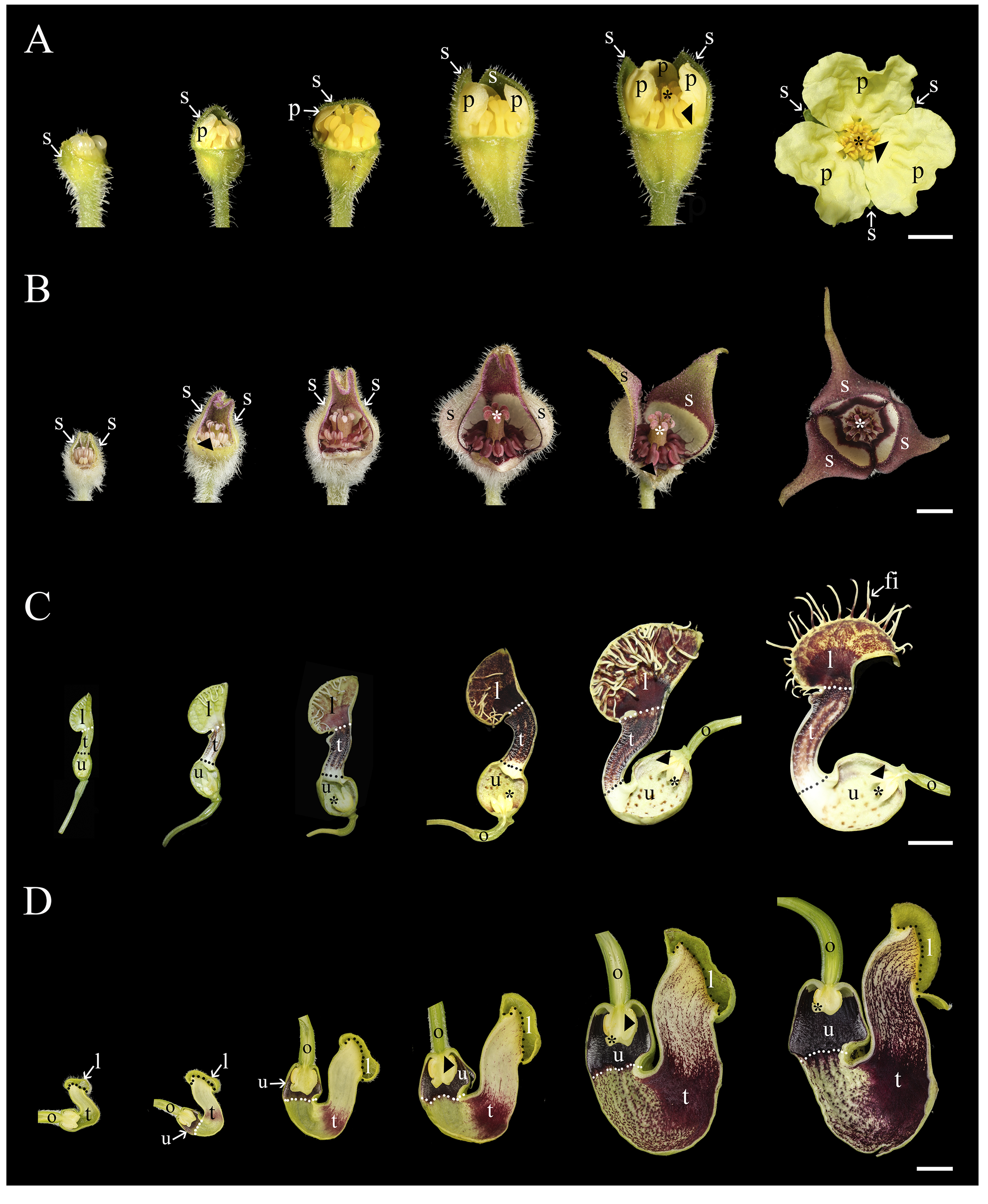

Supplement: Supplementary Figure 3 — Developmental stages sampled for RT-PCR of (A) Saruma henryi, (B) Asarum canadense, (C) Aristolochia fimbriata, (D) A. manshuriensis. l, limb; p, petal; s, sepal; t, tube; u, utricle. [file Image_3.TIF]

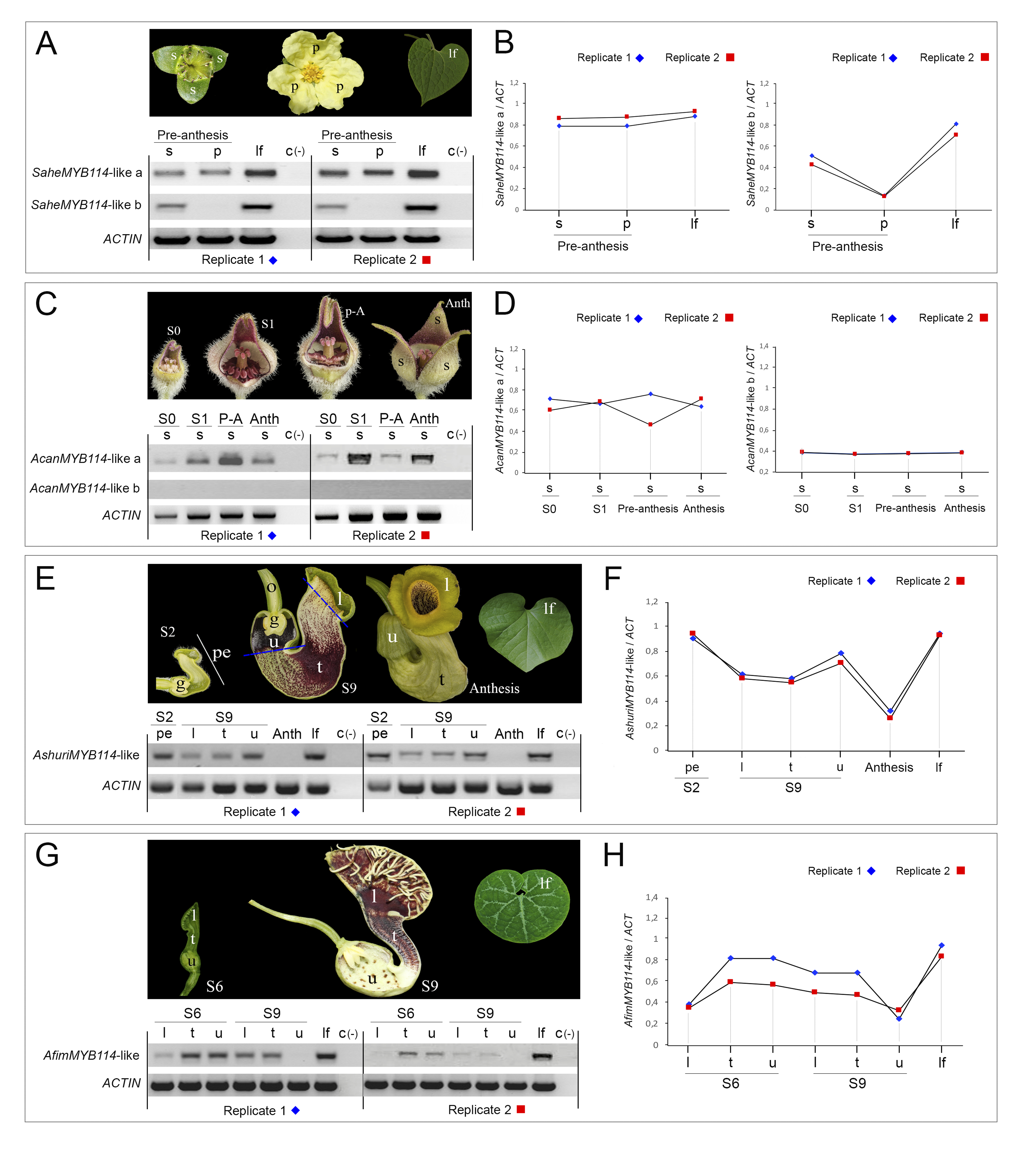

Supplement: Supplementary Figure 4 — Image J quantitative expression analysis based on the original electrophoresis images obtained by RT-PCR with an unprocessed background. [file Image_4.TIF]

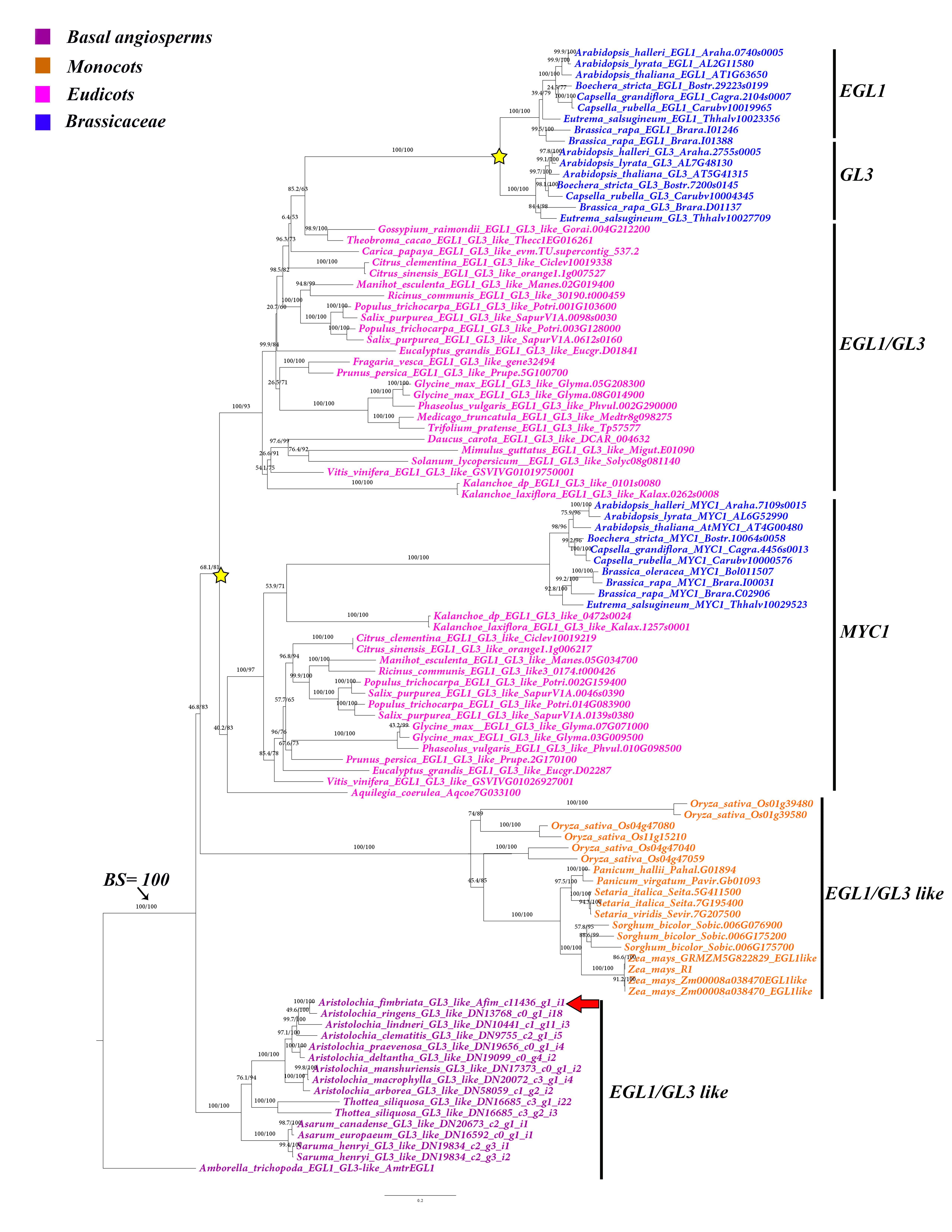

Supplement: Supplementary Figure 5 — Maximum likelihood analysis of the EGL1/GL3 bHLH genes across angiosperms. Yellow stars indicate large-scale duplication events. Color clades follow the conventions in the top left. The Aristolochia fimbriata homolog is pointed with a red arrow. [file Image_5.TIF]

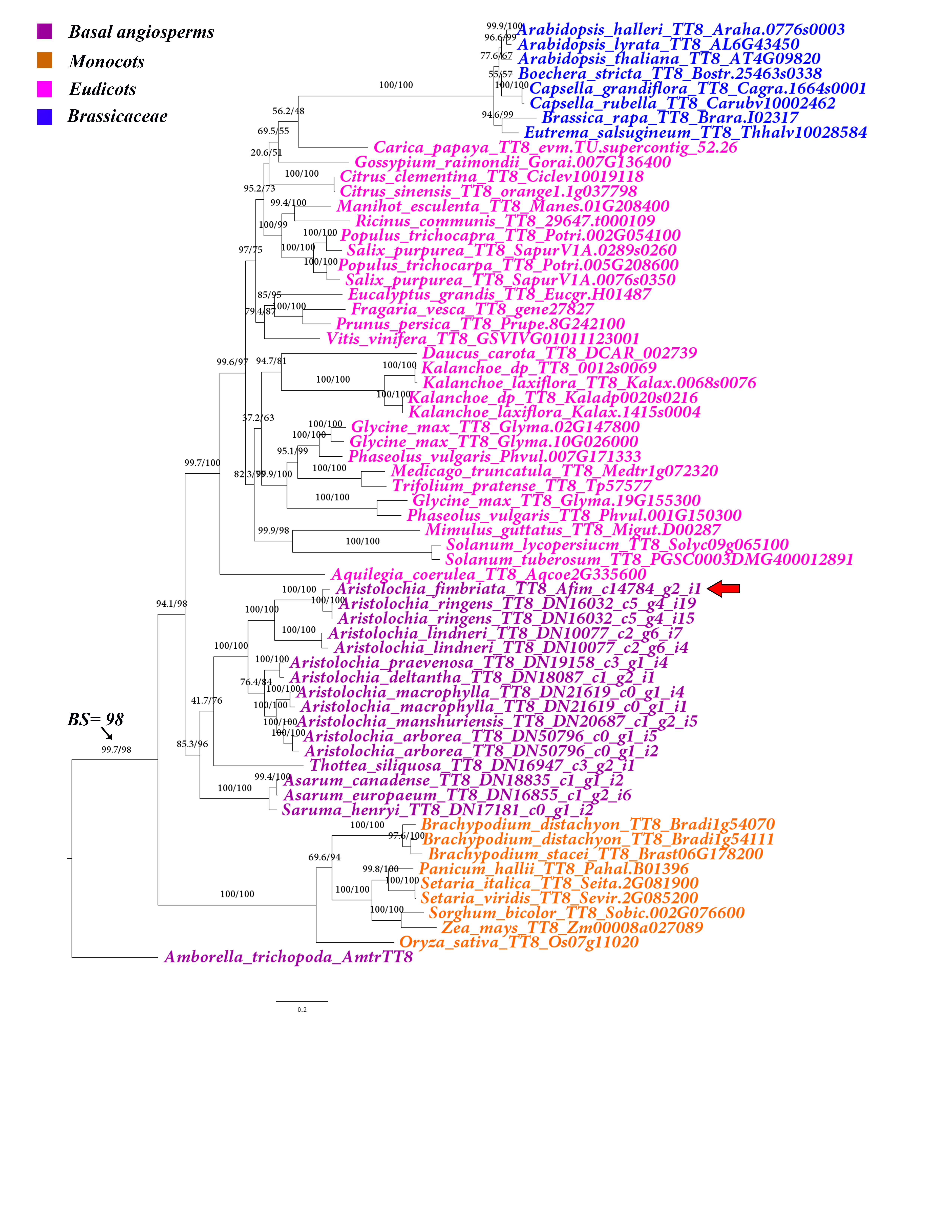

Supplement: Supplementary Figure 6 — Maximum likelihood analysis of the TT8 bHLH genes across angiosperms. Color clades follow the conventions in the top left. The Aristolochia fimbriata homolog is pointed with a red arrow. [file Image_6.TIF]

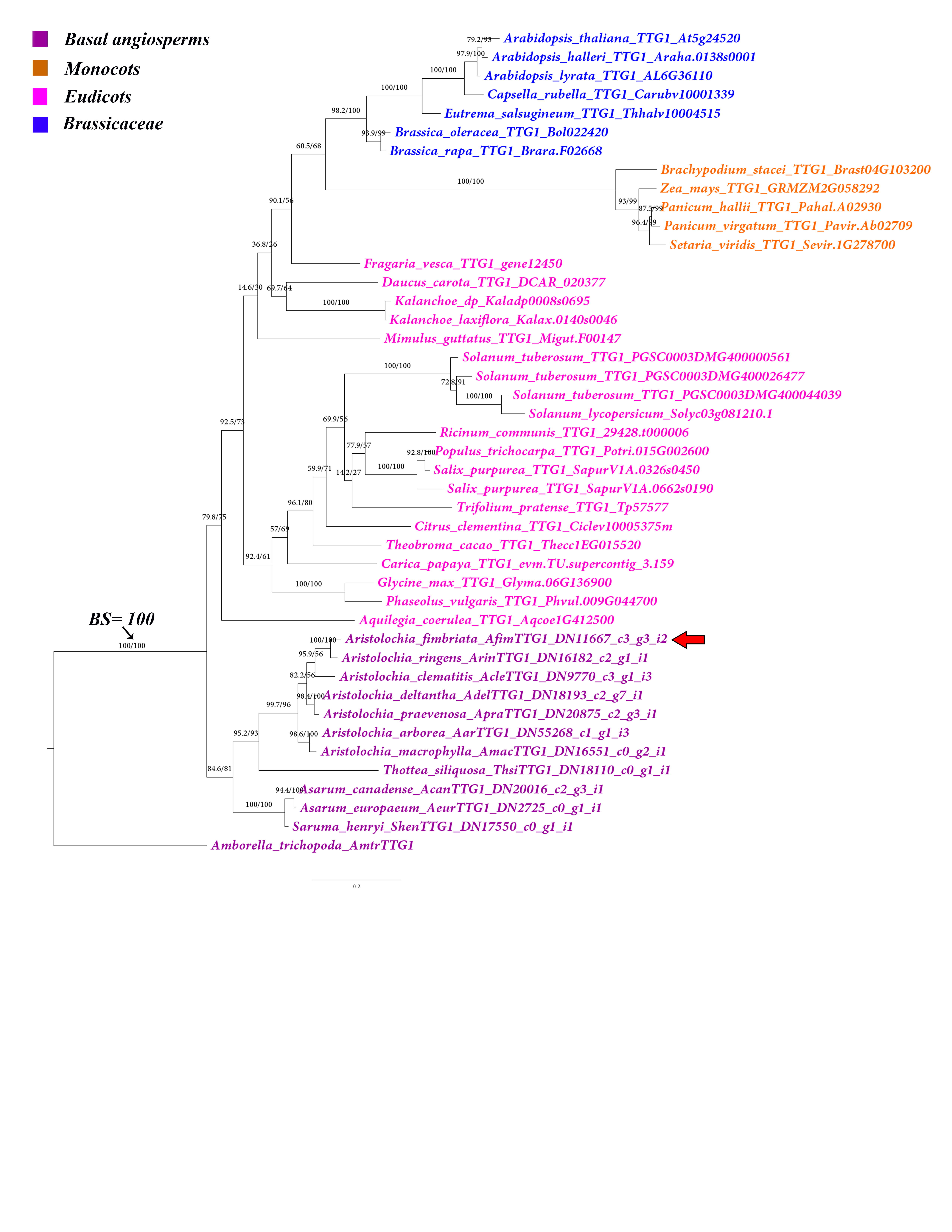

Supplement: Supplementary Figure 7 — Maximum likelihood analysis of the TTG1 WD40 genes across angiosperms. Color clades follow the conventions in the top left. The Aristolochia fimbriata homolog is pointed with a red arrow. [file Image_7.TIF]
