# Supplementary material for: Evolution of the Subgroup 6 R2R3-MYB Genes and Their Contribution to Floral Color in the Perianth-Bearing Piperales
Source: Front Plant Sci. 2021 Apr 9;12:633227. doi: 10.3389/fpls.2021.633227 (PMC8063865; doi:10.3389/fpls.2021.633227)
Supplement: Supplementary Table 1 — Names, abbreviations, codes, and original databases for all sequences included in the SG6 R2R3 MYB phylogenetic analyses. [file Table_1.DOCX]

**Supplementary Table 1.** Names, abbreviations and codes for all sequences included in the MYB R2R3 phylogenetic analysis. In purple are all new sequences isolated in this work.

| ***Species*** | **Original Code** | **Assigned name in the tree** | **Database** |
| --- | --- | --- | --- |
| *Allium cepa* | KX785130.1 | AlliumcepaMYB1 | NCBI |
| *Amborella trichopoda* | AmTr_v1.0_scaffold00048.187 | AmtrMYB114like | Phytozome |
| *Anthurium andraeanum* | KU726561 | AnanMYB2 | NCBI |
| *Antirrhinum majus* | DQ275529 | ROSEA1 | NCBI |
| *Antirrhinum majus* | DQ275530 | ROSEA2 | NCBI |
| *Antirrhinum majus* | DQ275531 | VENOSA | NCBI |
| *Aquilegia caerulea* | Aqcoe6G143800.1.p | Same as code | PTFDB |
| *Aquilegia caerulea* | Aqcoe6G143600.1.p | Same as code | PTFDB |
| *Aquilegia caerulea* | Aqcoe6G143700.1.p | Same as code | PTFDB |
| *Arabidopsis halleri* | Araha.13407s0001.1 | AhaMYB75 | Phytozome |
| *Arabidopsis halleri* | Araha.33597s0001.1 | AhaMYB90 | Phytozome |
| *Arabidopsis halleri* | Araha.6251s0001.1 | AhaMYB113 | Phytozome |
| *Arabidopsis lyrata* | AL1G66590.t1 | AlyMYB75 | Phytozome |
| *Arabidopsis lyrata* | AL2G24170.t1 | AlyMYB90 | Phytozome |
| *Arabidopsis lyrata* | AL2G24140.t1 | AlyMYB113 | Phytozome |
| *Arabidopsis lyrata* | AL2G24160.t1 | AlyMYB114 | Phytozome |
| *Arabidopsis thaliana* | AT1G56650.1 | AtMYB75 | TAIR |
| *Arabidopsis thaliana* | AT1G66390.1 | AtMYB90 | TAIR |
| *Arabidopsis thaliana* | AT1G66370.1 | AtMYB113 | TAIR |
| *Arabidopsis thaliana* | AT1G66380.1 | AtMYB114 | TAIR |
| *Aristolochia arborea* | TRINITY_DN47908_c0_g1_i3 | AarMYB114-like | MW125657 |
| *Aristolochia clematitis* | TRINITY_DN10310_c0_g1_i1 | AcleMYB114-like | MW125658 |
| *Aristolochia deltantha* | TRINITY_DN17287_c0_g11_i1 | AdelMYB114-like | MW125659 |
| *Aristolochia fimbriata* | c10687_g2_i2 | AfimMYB114-like | MW125662 |
| *Aristolochia lindneri* | TRINITY_DN10003_c3_g1_i3 | AlindMYB114-like | MW125660 |
| *Aristolochia macrophylla* | TRINITY_DN15194_c0_g1_i1 | AmacroMYB11like4-like | MW125655 |
| *Aristolochia manshuriensis* | TRINITY_DN11082_c0_g1_i1 | AshuriMYB114-like | MW125656 |
| *Aristolochia praevenosa* | TRINITY_DN19768_c2_g2_i11 | ApraMYB114-like | MW125661 |
| *Asarum canadense* | TRINITY_DN36779_c0_g1_i1 | AcanMYB114-like a | MW125649 |
| *Asarum canadense* | TRINITY_DN19985_c1_g4_i4 | AcanMYB114-like b | MW125650 |
| *Asarum europaeum* | TRINITY_DN15593_c4_g3_i1 | AeurMYB114-like a | MW125651 |
| *Asarum europaeum* | TRINITY_DN15894_c3_g3_i3 | AeurMYB114-like b | MW125652 |
| *Bocconia frutescens* | Amapola-117311 | BofrMYB114-like a | Evo Devo Transcriptomes |
| *Bocconia frutescens* | Amapola-56854 | BofrMYB114-like b | Evo Devo Transcriptomes |
| *Boechera stricta* | Bostr.26959s0279.1 | Same as code | PTFDB |
| *Boechera stricta* | Bostr.3125s0001.1 | Same as code | PTFDB |
| *Brassica nigra* | BraniMYB75 | Same as code | Phytozome |
| *Brassica oleracea* | Bole_XP_013627020.1 | Same as code | PTFDB |
| *Brassica oleracea* | Bole_XP_013627019.1 | Same as code | PTFDB |
| *Brassica oleracea* | Bole_XP_013613998.1 | Same as code | PTFDB |
| *Brassica oleracea* | Bole_XP_013591392.1 | Same as code | PTFDB |
| *Brassica oleracea* | Bole_XP_013590812.1 | Same as code | PTFDB |
| *Brassica oleracea* | Bol012528 | Same as code | Phytozome |
| *Brassica rapa* | Brara_XP_009135861.1 | Same as code | PTFDB |
| *Brassica rapa* | Brara_XP_009135860.1 | Same as code | PTFDB |
| *Brassica rapa* | Brara_XP_009105202.1 | Same as code | PTFDB |
| *Brassica rapa* | Brara_XP_009127485.1 | Same as code | PTFDB |
| *Brassica rapa* | Brara.C03920.1 | Same as code | Phytozome |
| *Brassica rapa* | Brara.B01656.1 | Same as code | Phytozome |
| *Brunfelsia australis* | BFR_TRINITY_DN60514_c0_g1_i2 | BrauMYB114-like | Evo Devo Transcriptomes |
| *Capsella grandiflora* | Cagra.2629s0007.1 | Same as code | PTFDB |
| *Capsella grandiflora* | Cagra.0463s0008.1 | Same as code | PTFDB |
| *Capsella rubella* | Carubv10022245m | Same as code | PTFDB |
| *Capsella rubella* | Carubv10020856m | Same as code | PTFDB |
| *Capsella rubella* | Carubv10020888m | Same as code | PTFDB |
| *Carica papaya* | evm.model.supercontig_2742.1 | Same as code | Phytozome |
| *Carica papaya* | evm.TU.contig_42663.2 | Same as code | Phytozome |
| *Cattleya trianae* | c21338_g1_i2 | CatrMYB114_like_a | Evo Devo Transcriptomes |
| *Cattleya trianae* | c21338_g1_i1 | CatrMYB114_like_b | Evo Devo Transcriptomes |
| *Chloranthus spicatus* | TRINITY_DN13388_c0_g1_i2 | ChspMYB114-like | Evo Devo Transcriptomes |
| *Daucus carota* | DCAR_010747 | Same as code | Phytozome |
| *Daucus carota* | DCAR_010745 | Same as code | Phytozome |
| *Daucus carota* | DCAR_010746 | Same as code | Phytozome |
| *Daucus carota* | DCAR_008994 | Same as code | Phytozome |
| *Epidendrum fimbriatum* | TRINITY_DN6914_c0_g1_i3 | EpfiMYB114_like_a | Evo Devo Transcriptomes |
| *Epidendrum fimbriatum* | TRINITY_DN2882_c0_g1_i14 | EpfiMYB114_like_b | Evo Devo Transcriptomes |
| *Epidendrum fimbriatum* | TRINITY_DN2882_c0_g1_i3 | EpfiMYB114_like_c | Evo Devo Transcriptomes |
| *Epidendrum fimbriatum* | TRINITY_DN2882_c0_g1_i6 | EpfiMYB114_like_d | Evo Devo Transcriptomes |
| *Eutrema salsugineum* | Thhalv10019041m | EusaMYB113/114-like | PTFDB |
| *Glycine max* | Glyma.19G025000.1.p | Same as code | PTFDB |
| *Glycine max* | Glyma.09G235300.1.p | Same as code | PTFDB |
| *Glycine max* | Glyma.18G261700.1.p | Same as code | PTFDB |
| *Glycine max* | Glyma.09G235100.1.p | Same as code | PTFDB |
| *Glycine max* | Glyma.18G262000.1.p | Same as code | PTFDB |
| *Glycine max* | Glyma.09G234900.1.p | Same as code | PTFDB |
| *Gossypium hirsutum* | NA | GohiMYB114-like a | Phytozome |
| *Gossypium hirsutum* | NA | GohiMYB114-like b | Phytozome |
| *Hedyosmum goudotianum* | TRINITY_DN48916_c3_g1_i2 | HegoMYB114_like_a | Evo Devo Transcriptomes |
| *Hedyosmum goudotianum* | TRINITY_DN85230_c0_g1_i1 | HegoMYB114_like_b | Evo Devo Transcriptomes |
| *Lilium hybrid* | AB534587 | LhMYB6 | NCBI |
| *Lilium hybrid* | AB534586 | LhMYB12 | NCBI |
| *Lilium regale* | LC021383 | LrMYB15 | NCBI |
| *Magnolia liliiflora* | KC794950.1 | MaliMYB114-like | NCBI |
| *Masdevallia coccinea* | TRINITY_DN22989_c0_g1_i1 | MacoMYB114_like_a | Evo Devo Transcriptomes |
| *Masdevallia coccinea* | TRINITY_DN18109_c0_g1_i1 | MacoMYB114_like_b | Evo Devo Transcriptomes |
| *Maxillaria aurea* | TRINITY_DN6902_c0_g1_i1 | MaauMYB114_like | Evo Devo Transcriptomes |
| *Medicago truncatula* | Medtr5g078800.1 | Same as code | PTFDB |
| *Medicago truncatula* | Medtr5g078860.1 | Same as code | PTFDB |
| *Medicago truncatula* | Medtr5g079220.1 | Same as code | PTFDB |
| *Medicago truncatula* | Medtr5g079290.1 | Same as code | PTFDB |
| *Medicago truncatula* | Medtr5g078950.1 | Same as code | PTFDB |
| *Medicago truncatula* | Medtr5g078910.1 | Same as code | PTFDB |
| *Medicago truncatula* | Medtr8g060940.1 | Same as code | PTFDB |
| *Medicago truncatula* | Medtr7g017260.1 | Same as code | PTFDB |
| *Medicago truncatula* | Medtr5g079120.1 | Same as code | PTFDB |
| *Medicago truncatula* | Medtr5g078930.1 | Same as code | PTFDB |
| *Miltoniopsis roezlii* | TRINITY_DN16712_c3_g4_i2 | MiroMYB114_like_a | Evo Devo Transcriptomes |
| *Miltoniopsis roezlii* | TRINITY_DN16712_c3_g4_i3 | MiroMYB114_like_b | Evo Devo Transcriptomes |
| *Mimulus guttatus* | Migut.H00278.1 | Same as code | Phytozome |
| *Mimulus guttatus* | Migut.L00458.1 | Same as code | Phytozome |
| *Nicotiana tabacum* | NitaMYB114-like | Same as code | PTFDB |
| *Nymphacea colorata* | XM_031637438.1 | NycoMYB114-like a | NCBI |
| *Nymphacea colorata* | XM_031618803.1 | NycoMYB114-like b | NCBI |
| *Oncidium v. twinkle* | TRINITY_DN22106_c0_g2_i1 | OntwMYB114_like | Evo Devo Transcriptomes |
| *Petunia hybrida* | AF166702 | AN2 | NCBI |
| *Petunia hybrida* | HQ428106 | AN4 | NCBI |
| *Petunia hybrida* | HQ116169 | PhDPL | NCBI |
| *Petunia hybrida* | HQ116170 | PhPHZ | NCBI |
| *Populus trichocarpa* | Potri.017G125600.1 | Same as code | PTFDB |
| *Populus trichocarpa* | Potri.017G125800.1 | Same as code | PTFDB |
| *Populus trichocarpa* | Potri.017G125700.1 | Same as code | PTFDB |
| *Populus trichocarpa* | Potri.017G125900.1 | Same as code | PTFDB |
| *Populus trichocarpa* | Potri.017G126000.1 | Same as code | PTFDB |
| *Prunus persica* | Prupe.3G163300.1 | Same as code | Phytozome |
| *Prunus persica* | Prupe.3G163100.1 | Same as code | Phytozome |
| *Prunus persica* | Prupe.3G163000.1 | Same as code | Phytozome |
| *Rhyncholaelicocattleya* | MN420461 | RcPAP1 | NCBI |
| *Rhyncholaelicocattleya* | MN420462 | RcPAP2 | NCBI |
| *Sarcandra chloranthoides* | TRINITY_DN12909_c2_g1_i3 | SachMYB114_like_a | Evo Devo Transcriptomes |
| *Sarcandra chloranthoides* | TRINITY_DN12909_c2_g1_i1 | SachMYB114_like_b | Evo Devo Transcriptomes |
| *Saruma henryi* | TRINITY_DN14090_c0_g1_i1 | SaheMYB114-like a | MW125647 |
| *Saruma henryi* | TRINITY_DN21079_c3_g5_i2 | SaheMYB114-like b | MW125648 |
| *Solanum lycopersicum* | Solyc10g086290.1.1 | Same as code | PTFDB |
| *Solanum lycopersicum* | Solyc10g086250.1.1 | Same as code | PTFDB |
| *Solanum lycopersicum* | Solyc10g086260.1.1 | Same as code | PTFDB |
| *Solanum lycopersicum* | Solyc10g086270.1.1 | Same as code | PTFDB |
| *Solanum melongena* | Sme2.5_05099.1_g00002.1 | Same as code | PTFDB |
| *Solanum pennellii* | Sopen10g035680.1 | Same as code | PTFDB |
| *Solanum pennellii* | Sopen10g035640.1 | Same as code | PTFDB |
| *Solanum pennellii* | Sopen10g035650.1 | Same as code | PTFDB |
| *Solanum pennellii* | Sopen10g035660.1 | Same as code | PTFDB |
| *Solanum pimpinellifolium* | Sopim10g086290.0.1 | Same as code | PTFDB |
| *Solanum pimpinellifolium* | Sopim10g086250.0.1 | Same as code | PTFDB |
| *Solanum pimpinellifolium* | Sopim10g086260.0.1 | Same as code | PTFDB |
| *Solanum pimpinellifolium* | Sopim10g086270.0.1 | Same as code | PTFDB |
| *Solanum tuberosum* | StMYB134 | Same as code | Zhao et al., 2013 |
| *Solanum tuberosum* | StMYB137 | Same as code | Zhao et al., 2013 |
| *Streptosolen jamesonii* | STE_TRINITY_DN26712_c0_g2_i1 | StjaMYB114-like a | Evo Devo Transcriptomes |
| *Streptosolen jamesonii* | STE_TRINITY_DN252_c0_g1_i1 | StjaMYB114-like b | Evo Devo Transcriptomes |
| *Theobroma cacao* | Thecc1EG019192t1 | ThcaMYB114-like | Phytozome |
| *Thottea siliquosa* | TRINITY_DN16796_c2_g1_i1 | ThsiMYB114-like-a | MW125653 |
| *Thottea siliquosa* | TRINITY_DN16796_c2_g1_i2 | ThsiMYB114-b | MW125654 |
| *Tolumnia v. Cherry* | TRINITY_DN20692_c0_g1_i5 | TolMYB114_like_a | Evo Devo Transcriptomes |
| *Tolumnia v. Cherry* | TRINITY_DN20692_c0_g1_i6 | TolMYB114_like_b | Evo Devo Transcriptomes |
| *Tropaeolum longifolium* | Trope_TRINITY_DN17254_c0_g1_i4 | TrloMYB114-like a | Evo Devo Transcriptomes |
| *Tropaeolum longifolium* | Trope_TRINITY_DN17254_c0_g1_i5 | TrloMYB114-like b | Evo Devo Transcriptomes |
| *Tropaeolum longifolium* | Trope_TRINITY_DN17254_c0_g1_i1 | TrloMYB114-like c | Evo Devo Transcriptomes |
| *Tropaeolum majus* | TRINITY_ DN45296_c0_g2_i1 | TrmaMYB114like a | Evo Devo Transcriptomes |
| *Tropaeolum majus* | TRINITY__DN45296_c0_g1_i1 | TrmaMYB114like b | Evo Devo Transcriptomes |
| *Vitis vinifera* | GSVIVT01022657001 | Same as code | PTFDB |
| *Vitis vinifera* | GSVIVT01022661001 | Same as code | PTFDB |
| *Vitis vinifera* | GSVIVT01022659001 | Same as code | PTFDB |
| *Vitis vinifera* | GSVIVT01022656001 | Same as code | PTFDB |
| *Vitis vinifera* | GSVIVT01022654001 | Same as code | PTFDB |
| *Vitis vinifera* | GSVIVT01022664001 | Same as code | PTFDB |
| *Vitis vinifera* | GSVIVT01030819001 | Same as code | PTFDB |
| *Vitis vinifera* | GSVIVT01030822001_2 | Same as code | PTFDB |
