# Supplementary material for: Evolution of the Subgroup 6 R2R3-MYB Genes and Their Contribution to Floral Color in the Perianth-Bearing Piperales
Source: Front Plant Sci. 2021 Apr 9;12:633227. doi: 10.3389/fpls.2021.633227 (PMC8063865; doi:10.3389/fpls.2021.633227)
Supplement: Supplementary Table 2 — Name, sequence, and melting temperature of the primers used during RT-PCR analyses. [file Table_2.DOCX]

| **Primer name** | **Primer sequence** | **Tm** |
| --- | --- | --- |
| Sahe79_Fwd | CGTGGACACAAGAAGAAGACA | 63.4 |
| Sahe79_Rev | GAGTTTGAAGGGTGGCTTTCTA | 63.6 |
| AfimMYB75_Fwd | TGAATAGGTGCCGCAAAA | 63.3 |
| AfimMYB75_Rev | AGAAGATGAAACCCATCAAG | 58.7 |
| Aeur94_Fwd | ACGTGAGAAAAGGGTCATGG | 64.3 |
| Aeur94_Rev | ATATGAGGTGGCAGATAATTCA | 60.3 |
| Aeur93_Fwd | CCCCCAAATGTAAGAAAAGG | 62.4 |
| Aeur93_Rev | AAGATGATTTGCTGTCTGGG | 62.1 |
| Ashuri82_Fwd | GAAGAAGACCTCCTGCTCAG | 61.2 |
| Ashuri82_Rev | CAGTGACACATCCCATGGAT | 62.5 |
| Amacro94_Fwd | TGACTTTGCAGTCGATGAGG | 64.5 |
| Amacro94_Rev | AAAAGCTTCAGAGATGGATC | 58 |
| Aar08_Fwd | GAAGAAGACCTCCTGCTCAG | 61.2 |
| Aar08_Rev | ATCGAGTGACACAGCCCA | 63.7 |

**Supplementary Table 2**. Name, sequence, and melting temperature of the primers used during RT-PCR analyses.
