# Supplementary material for: Evolution of the Subgroup 6 R2R3-MYB Genes and Their Contribution to Floral Color in the Perianth-Bearing Piperales
Source: Front Plant Sci. 2021 Apr 9;12:633227. doi: 10.3389/fpls.2021.633227 (PMC8063865; doi:10.3389/fpls.2021.633227)
Supplement: Supplementary Table 3 — De novo assembly of the floral transcriptomes of Aristolochia fimbriata at two different developmental stages. [file Table_3.DOCX]

**Supplementary Table 3.** *De novo* assembly of the floral transcriptomes of *Aristolochia fimbriata* at two different developmental stages.

| **Sample** | **Transcriptome assembly statistics (TRINITY)** |
| --- | --- |
| ***Aristolochia fimbriata***  **(Limb S6)** | Total length of sequence: 173083699 bp  Total number of sequences: 96132  Average contig length is: 1800 bp  Largest contig: 17324 bp  Shortest contig: 201 bp  N25 stats: 25% of total sequence length is contained in the 8035 sequences >= 4055 bp  N50 stats: 50% of total sequence length is contained in the 21058 sequences >= 2766 bp  N75 stats: 75% of total sequence length is contained in the 40487 sequences >= 1768 bp  Total GC count: 73740853 bp  GC %: 42.60 %  Number of Ns: 0  Ns %: 0.00 % |
| ***Aristolochia fimbriata***  **(Tube S6)** | Total length of sequence: 88849014 bp  Total number of sequences: 66217  Average contig length is: 1341 bp  Largest contig: 13435 bp  Shortest contig: 201 bp  N25 stats: 25% of total sequence length is contained in the 6413 sequences >= 2707 bp  N50 stats: 50% of total sequence length is contained in the 16174 sequences >= 1946 bp  N75 stats: 75% of total sequence length is contained in the 29968 sequences >= 1305 bp  Total GC count: 38610430 bp  GC %: 43.46 %  Number of Ns: 0  Ns %: 0.00 % |
| ***Aristolochia fimbriata***  **(Utricle S6)** | Total length of sequence: 184564520 bp  Total number of sequences: 101267  Average contig length is: 1822 bp  Largest contig: 17241 bp  Shortest contig: 201 bp  N25 stats: 25% of total sequence length is contained in the 8397 sequences >= 4131 bp  N50 stats: 50% of total sequence length is contained in the 22175 sequences >= 2779 bp  N75 stats: 75% of total sequence length is contained in the 42823 sequences >= 1777 bp  Total GC count: 78795454 bp  GC %: 42.69 %  Number of Ns: 0  Ns %: 0.00 % |
| ***Aristolochia fimbriata***  **(Limb S9)** | Total length of sequence: 124735043 bp  Total number of sequences: 90543  Average contig length is: 1377 bp  Largest contig: 15080 bp  Shortest contig: 201 bp  N25 stats: 25% of total sequence length is contained in the 6769 sequences >= 3431 bp  N50 stats: 50% of total sequence length is contained in the 18222 sequences >= 2212 bp  N75 stats: 75% of total sequence length is contained in the 36313 sequences >= 1311 bp  Total GC count: 53347519 bp  GC %: 42.77 %  Number of Ns: 0  Ns %: 0.00 % |
| ***Aristolochia fimbriata***  **(Tube S9)** | Total length of sequence: 150478482 bp  Total number of sequences: 123439  Average contig length is: 1219 bp  Largest contig: 14074 bp  Shortest contig: 201 bp  N25 stats: 25% of total sequence length is contained in the 9846 sequences >= 2841 bp  N50 stats: 50% of total sequence length is contained in the 26269 sequences >= 1877 bp  N75 stats: 75% of total sequence length is contained in the 52141 sequences >= 1101 bp  Total GC count: 64319073 bp  GC %: 42.74 %  Number of Ns: 0  Ns %: 0.00 % |
| ***Aristolochia fimbriata***  **(Utricle S9)** | Total length of sequence: 145574976 bp  Total number of sequences: 123214  Average contig length is: 1181 bp  Largest contig: 16531 bp  Shortest contig: 201 bp  N25 stats: 25% of total sequence length is contained in the 9698 sequences >= 2797 bp  N50 stats: 50% of total sequence length is contained in the 26012 sequences >= 1812 bp  N75 stats: 75% of total sequence length is contained in the 52079 sequences >= 1054 bp  Total GC count: 62305178 bp  GC %: 42.80 %  Number of Ns: 0  Ns %: 0.00 % |
