# Supplementary material for: Evolution of the Subgroup 6 R2R3-MYB Genes and Their Contribution to Floral Color in the Perianth-Bearing Piperales
Source: Front Plant Sci. 2021 Apr 9;12:633227. doi: 10.3389/fpls.2021.633227 (PMC8063865; doi:10.3389/fpls.2021.633227)
Supplement: Supplementary Table 4 — Expression of the candidate genes included in this study. Values correspond to the TPMs. [file Table_4.DOCX]

| **Supplementary Table 4.** Expression of the candidate genes included in this study. Values shown here correspond to the TPMs. | | | | | | | | | | | | | | |
| --- | --- | --- | --- | --- | --- | --- | --- | --- | --- | --- | --- | --- | --- | --- |
|  |  | | |  |  | |  |  | | |  | |  |  |
|  | |  |  | | | **TPMs (transcripts per million)** | | | | | | |  |  |
| **Gene name** | | **Contig** | **Gene Family** | | | **Limb S6** | **Limb S9** | **Tube S6** | **Tube S9** | **Utricle S6** | | **Utricle S9** |  |  |
| *AfimCHS* | | TRINITY_DN9802_c3_g1_i2 | [Chalcone/stilbene synthases family](https://www.uniprot.org/uniprot/?query=family:%22thiolase-like+superfamily.+Chalcone%2Fstilbene+synthases+family%22&sort=score)^1^ | | | 2533,3 | 23128,9 | 3429,0 | 12335,0 | 1731,2 | | 1499,0 |  |  |
| *AfimCHI* | | TRINITY_DN10005_c1_g2_i2 | [Chalcone isomerase family](https://www.uniprot.org/uniprot/?query=family:%22chalcone+isomerase+family%22&sort=score) ^1^ | | | 395,2 | 222,7 | 544,9 | 419,3 | 335,1 | | 3029,7 |  |  |
| *AfimANS* | | TRINITY_DN11096_c1_g3_i1 | [Iron/ascorbate-dependent oxidoreductase family](https://www.uniprot.org/uniprot/?query=family:%22iron%2Fascorbate-dependent+oxidoreductase+family%22&sort=score) ^1^ | | | 65,7 | 1713,1 | 559,0 | 5403,2 | 21,9 | | 3837,7 |  |  |
| *AfimF3H* | | TRINITY_DN10618_c3_g5_i1 | [Iron/ascorbate-dependent oxidoreductase family](https://www.uniprot.org/uniprot/?query=family:%22iron%2Fascorbate-dependent+oxidoreductase+family%22&sort=score) ^1^ | | | 2369,1 | 4858,2 | 4876,6 | 10242,0 | 2158,9 | | 7249,9 |  |  |
| *AfimBZ1* | | TRINITY_DN8920_c2_g6_i4 | [UDP-glycosyltransferase family](https://www.uniprot.org/uniprot/?query=family:%22UDP-glycosyltransferase+family%22&sort=score) ^1^ | | | 179,6 | 155,1 | 133,9 | 137,0 | 148,7 | | 288,3 |  |  |
| *AfimDFR* | | TRINITY_DN9918_c0_g1_i8 | [NAD(P)-dependent epimerase/dehydratase family](https://www.uniprot.org/uniprot/?query=family:%22NAD%28P%29-dependent+epimerase%2Fdehydratase+family%22&sort=score) ^1^ | | | 16,9 | 40,5 | 8,2 | 1815,9 | 25,5 | | 1957,6 |  |  |
| *AfimMYB114-like* | | TRINITY_DN9480_c1_g5_i2 | R2R3-MYB ^2^ | | | 664,2 | 231,5 | 590,8 | 321,6 | 613,0 | | 154,8 |  |  |
| *AfimTT8* | | TRINITY_DN9239_c2_g1_i10 | bHLH ^2^ | | | 0,0 | 7,9 | 24,1 | 6,0 | 6,7 | | 3,2 |  |  |
| *AfimGL3* | | TRINITY_DN10530_c1_g1_i16 | bHLH ^2^ | | | 41,6 | 21,1 | 61,2 | 0,0 | 14,1 | | 30,7 |  |  |
| *AfimTTG1* | | TRINITY_DN11667_c3_g3_i2 | WD-40 Repeat Protein ^2^ | | | 1257,4 | 1918,5 | 772,4 | 4067,0 | 900,3 | | 2430,0 |  |  |

^1^ Classification from UniProt database.

^2^ Classification from TAIR database.
