# Supplementary material for: Evolution of the Subgroup 6 R2R3-MYB Genes and Their Contribution to Floral Color in the Perianth-Bearing Piperales
Source: Front Plant Sci. 2021 Apr 9;12:633227. doi: 10.3389/fpls.2021.633227 (PMC8063865; doi:10.3389/fpls.2021.633227)
Supplement: Supplementary Table 5 — Names, abbreviations, codes, and original databases for all sequences included in the bHLH phylogenetic analyses. [file Table_5.DOCX]

**Supplementary Table 5.** Names, abbreviations and codes for all sequences included in the bHLH phylogenetic analysis. In purple are all new sequences isolated in this work.

| **Species** | **Original Code** | **Assigned name in the tree** | **Database** |
| --- | --- | --- | --- |
| *Amborella_trichopoda* | AmtrTT8 | AmtrTT8 | Phytozome |
| *Aquilegia_coerulea* | Aqcoe2G335600 | Aquilegia_coerulea_TT8_ | Phytozome |
| *Arabidopsis_halleri* | Araha.0776s0003 | Arabidopsis_halleri_TT8_ | Phytozome |
| *Arabidopsis_lyrata* | AL6G43450 | Arabidopsis_lyrata_TT8_ | Phytozome |
| *Arabidopsis_thaliana* | AT4G09820 | Arabidopsis_thaliana_TT8_ | Phytozome |
| *Aristolochia_arborea* | DN50796_c0_g1_i2 | Aristolochia_arborea_TT8_a | Evo Devo Transcriptomes |
| *Aristolochia_arborea* | DN50796_c0_g1_i5 | Aristolochia_arborea_TT8_b | Evo Devo Transcriptomes |
| *Aristolochia_deltantha* | DN18087_c1_g2_i1 | Aristolochia_deltantha_TT8_ | Evo Devo Transcriptomes |
| *Aristolochia_fimbriata* | Afim_c14784_g2_i1 | Aristolochia_fimbriata_TT8_ | Evo Devo Transcriptomes |
| *Aristolochia_lindneri* | DN10077_c2_g6_i4 | Aristolochia_lindneri_TT8_ | Evo Devo Transcriptomes |
| *Aristolochia_lindneri* | DN10077_c2_g6_i7 | Aristolochia_lindneri_TT8_ | Evo Devo Transcriptomes |
| *Aristolochia_macrophylla* | DN21619_c0_g1_i1 | Aristolochia_macrophylla_TT8_a | Evo Devo Transcriptomes |
| *Aristolochia_macrophylla* | DN21619_c0_g1_i4 | Aristolochia_macrophylla_TT8_b | Evo Devo Transcriptomes |
| *Aristolochia_manshuriensis* | DN20687_c1_g2_i5 | Aristolochia_manshuriensis_TT8_ | Evo Devo Transcriptomes |
| *Aristolochia_praevenosa* | DN19158_c3_g1_i4 | Aristolochia_praevenosa_TT8_ | Evo Devo Transcriptomes |
| *Aristolochia_ringens* | DN16032_c5_g4_i15 | Aristolochia_ringens_TT8_a | Evo Devo Transcriptomes |
| *Aristolochia_ringens* | DN16032_c5_g4_i19 | Aristolochia_ringens_TT8_b | Evo Devo Transcriptomes |
| *Asarum_canadense* | DN18835_c1_g1_i2 | Asarum_canadense_TT8_ | Evo Devo Transcriptomes |
| *Asarum_europaeum* | DN16855_c1_g2_i6 | Asarum_europaeum_TT8_ | Evo Devo Transcriptomes |
| *Boechera_stricta* | Bostr.25463s0338 | Boechera_stricta_TT8_ | Phytozome |
| *Brachypodium_distachyon* | Bradi1g54070 | Brachypodium_distachyon_TT8_a | Phytozome |
| *Brachypodium_distachyon* | Bradi1g54111 | Brachypodium_distachyon_TT8_b | Phytozome |
| *Brachypodium_stacei* | Brast06G178200 | Brachypodium_stacei_TT8 | Phytozome |
| *Brassica_rapa* | Brara.I02317 | Brassica_rapa_TT8 | Phytozome |
| *Capsella_grandiflora* | Cagra.1664s0001 | Capsella_grandiflora_TT8_ | Phytozome |
| *Capsella_rubella* | Carubv10002462 | Capsella_rubella_TT8_ | Phytozome |
| *Carica_papaya* | evm.TU.supercontig_52.26 | Carica_papaya_TT8 | Phytozome |
| *Citrus_clementina* | Ciclev10019118 | Citrus_clementina_TT8_ | Phytozome |
| *Citrus_sinensis* | orange1.1g037798 | Citrus_sinensis_TT8_ | Phytozome |
| *Daucus_carota* | DCAR_002739 | Daucus_carota_TT8_ | Phytozome |
| *Eucalyptus_grandis* | Eucgr.H01487 | Eucalyptus_grandis_TT8_ | Phytozome |
| *Eutrema_salsugineum* | Thhalv10028584 | Eutrema_salsugineum_TT8_ | Phytozome |
| *Fragaria_vesca* | gene27827 | Fragaria_vesca_TT8_ | Phytozome |
| *Glycine_max* | Glyma.02G147800 | Glycine_max_TT8_a | Phytozome |
| *Glycine_max* | Glyma.10G026000 | Glycine_max_TT8_b | Phytozome |
| *Glycine_max* | Glyma.19G155300 | Glycine_max_TT8_c | Phytozome |
| *Gossypium_raimondii* | Gorai.007G136400 | Gossypium_raimondii_TT8 | Phytozome |
| *Kalanchoe_dp_* | 0012s0069 | Kalanchoe_dp_TT8_a | Phytozome |
| *Kalanchoe_dp* | Kaladp0020s0216 | Kalanchoe_dp_TT8_b | Phytozome |
| *Kalanchoe_laxiflora* | Kalax.1415s0004 | Kalanchoe_laxiflora_TT8_a | Phytozome |
| *Kalanchoe_laxiflora* | Kalax.0068s0076 | Kalanchoe_laxiflora_TT8_b | Phytozome |
| *Manihot_esculenta* | Manes.01G208400 | Manihot_esculenta_TT8_ | Phytozome |
| *Medicago_truncatula* | Medtr1g072320 | Medicago_truncatula_TT8_ | Phytozome |
| *Mimulus_guttatus* | Migut.D00287 | Mimulus_guttatus_TT8_ | Phytozome |
| *Oryza_sativa* | Os07g11020 | Oryza_sativa_TT8_ | Phytozome |
| *Panicum_hallii* | Pahal.B01396 | Panicum_hallii_TT8_ | Phytozome |
| *Phaseolus_vulgaris* | Phvul.007G171333 | Phaseolus_vulgaris_TT8_a | Phytozome |
| *Phaseolus_vulgaris* | Phvul.001G150300 | Phaseolus_vulgaris_TT8_b | Phytozome |
| *Populus_trichocarpa* | Potri.002G054100 | Populus_trichocarpa_TT8_a | Phytozome |
| *Populus_trichocarpa* | Potri.005G208600 | Populus_trichocarpa_TT8_b | Phytozome |
| *Prunus_persica* | Prupe.8G242100 | Prunus_persica_TT8 | Phytozome |
| *Ricinus_communis* | 29647.t000109 | Ricinus_communis_TT8_ | Phytozome |
| *Salix_purpurea* | SapurV1A.0076s0350 | Salix_purpurea_TT8_a | Phytozome |
| *Salix_purpurea* | SapurV1A.0289s0260 | Salix_purpurea_TT8_b | Phytozome |
| *Saruma_henryi* | DN17181_c0_g1_i2 | Saruma_henryi_TT8_ | Evo Devo Transcriptomes |
| *Setaria_italica* | Seita.2G081900 | Setaria_italica_TT8_ | Phytozome |
| *Setaria_viridis* | Sevir.2G085200 | Setaria_viridis_TT8_ | Phytozome |
| *Solanum_lycopersicum* | Solyc09g065100 | Solanum_lycopersiucm_TT8_ | Phytozome |
| *Solanum_tuberosum* | PGSC0003DMG400012891 | Solanum_tuberosum_TT8_ | Phytozome |
| *Sorghum_bicolor* | Sobic.002G076600 | Sorghum_bicolor_TT8_ | Phytozome |
| *Thottea_siliquosa* | DN16947_c3_g2_i1 | Thottea_siliquosa_TT8 | Phytozome |
| *Trifolium_pratense* | Tp57577 | Trifolium_pratense_TT8_ | Phytozome |
| *Vitis_vinifera* | GSVIVG01011123001 | Vitis_vinifera_TT8_ | Phytozome |
| *Zea_mays* | Zm00008a027089 | Zea_mays_TT8_ | Phytozome |

| ***Species*** | **Original Code** | **Assigned name in the tree** | **Database** |
| --- | --- | --- | --- |
| *Amborella_trichopoda* | AmtrEGL1 | Amborella_trichopoda_GL3-like | Phytozome |
| *Aquilegia_coerulea* | Aqcoe7G033100 | Aquilegia_coerulea_GL3_like | Phytozome |
| *Arabidopsis_halleri* | Araha.0740s0005 | Arabidopsis_halleri_EGL1 | Phytozome |
| *Arabidopsis_halleri* | Araha.2755s0005 | Arabidopsis_halleri_GL3 | Phytozome |
| *Arabidopsis_halleri* | Araha.7109s0015 | Arabidopsis_halleri_MYC1 | Phytozome |
| *Arabidopsis_lyrata* | AL2G11580 | Arabidopsis_lyrata_EGL1 | Phytozome |
| *Arabidopsis_lyrata* | AL7G48130 | Arabidopsis_lyrata_GL3 | Phytozome |
| *Arabidopsis_lyrata* | AL6G52990 | Arabidopsis_lyrata_MYC1 | Phytozome |
| *Arabidopsis_thaliana* | AT4G00480 | Arabidopsis_thaliana_AtMYC1 | Phytozome |
| *Arabidopsis_thaliana* | AT1G63650 | Arabidopsis_thaliana_EGL1 | Phytozome |
| *Arabidopsis_thaliana* | AT5G41315 | Arabidopsis_thaliana_GL3 | Phytozome |
| *Aristolochia_arborea* | DN58059_c1_g2_i2 | Aristolochia_arborea_GL3_like_ | Evo Devo Transcriptomes |
| *Aristolochia_clematitis* | DN9755_c2_g1_i5 | Aristolochia_clematitis_GL3_like | Evo Devo Transcriptomes |
| *Aristolochia_deltantha* | DN19099_c0_g4_i2 | Aristolochia_deltantha_GL3_like_ | Evo Devo Transcriptomes |
| *Aristolochia_fimbriata* | Afim_c11436_g1_i1 | Aristolochia_fimbriata_GL3_like_ | Evo Devo Transcriptomes |
| *Aristolochia_lindneri* | DN10441_c1_g11_i3 | Aristolochia_lindneri_GL3_like_ | Evo Devo Transcriptomes |
| *Aristolochia_macrophylla* | DN20072_c3_g1_i4 | Aristolochia_macrophylla_GL3_like_ | Evo Devo Transcriptomes |
| *Aristolochia_manshuriensis* | DN17373_c0_g1_i2 | Aristolochia_manshuriensis_GL3_like_ | Evo Devo Transcriptomes |
| *Aristolochia_praevenosa* | DN19656_c0_g1_i4 | Aristolochia_praevenosa_GL3_like_ | Evo Devo Transcriptomes |
| *Aristolochia_ringens* | DN13768_c0_g1_i18 | Aristolochia_ringens_GL3_like_ | Evo Devo Transcriptomes |
| *Asarum_canadense* | DN20673_c2_g1_i1 | Asarum_canadense_GL3_like_ | Evo Devo Transcriptomes |
| *Asarum_europaeum* | DN16592_c0_g1_i1 | Asarum_europaeum_GL3_like_ | Evo Devo Transcriptomes |
| *Boechera_stricta* | Bostr.29223s0199 | Boechera_stricta_EGL1_ | Phytozome |
| *Boechera_stricta* | Bostr.7200s0145 | Boechera_stricta_GL3_ | Phytozome |
| *Boechera_stricta* | Bostr.10064s0058 | Boechera_stricta_MYC1_ | Phytozome |
| *Brassica_oleracea* | Bol011507 | Brassica_oleracea_MYC1_ | Phytozome |
| *Brassica_rapa* | Brara.I01246 | Brassica_rapa_EGL1_a | Phytozome |
| *Brassica_rapa* | Brara.I01388 | Brassica_rapa_EGL1_b | Phytozome |
| *Brassica_rapa* | Brara.D01137 | Brassica_rapa_GL3_ | Phytozome |
| *Brassica_rapa* | Brara.C02906 | Brassica_rapa_MYC1_a | Phytozome |
| *Brassica_rapa* | Brara.I00031 | Brassica_rapa_MYC1_b | Phytozome |
| *Capsella_grandiflora* | Cagra.2104s0007 | Capsella_grandiflora_EGL1_ | Phytozome |
| *Capsella_grandiflora* | Cagra.4456s0013 | Capsella_grandiflora_MYC1_ | Phytozome |
| *Capsella_rubella* | Carubv10019965 | Capsella_rubella_EGL1_ | Phytozome |
| *Capsella_rubella* | Carubv10004345 | Capsella_rubella_GL3_ | Phytozome |
| *Capsella_rubella* | Carubv10000576 | Capsella_rubella_MYC1_ | Phytozome |
| *Carica_papaya* | evm.TU.supercontig_537.2 | Carica_papaya_EGL1_GL3_like_ | Phytozome |
| *Citrus_clementina* | Ciclev10019219 | Citrus_clementina_EGL1_GL3_like_a | Phytozome |
| *Citrus_clementina* | Ciclev10019338 | Citrus_clementina_EGL1_GL3_like_b | Phytozome |
| *Citrus_sinensis* | orange1.1g006217 | Citrus_sinensis_EGL1_GL3_like_ | Phytozome |
| *Citrus_sinensis* | orange1.1g007527 | Citrus_sinensis_EGL1_GL3_like_ | Phytozome |
| *Daucus_carota* | DCAR_004632 | Daucus_carota_EGL1_GL3_like_ | Phytozome |
| *Eucalyptus_grandis* | Eucgr.D01841 | Eucalyptus_grandis_EGL1_GL3_like_ | Phytozome |
| *Eucalyptus_grandis* | Eucgr.D02287 | Eucalyptus_grandis_EGL1_GL3_like_ | Phytozome |
| *Eutrema_salsugineum* | Thhalv10023356 | Eutrema_salsugineum_EGL1_ | Phytozome |
| *Eutrema_salsugineum* | Thhalv10027709 | Eutrema_salsugineum_GL3_ | Phytozome |
| *Eutrema_salsugineum* | Thhalv10029523 | Eutrema_salsugineum_MYC1_ | Phytozome |
| *Fragaria_vesca* | gene32494 | Fragaria_vesca_EGL1_GL3_like_ | Phytozome |
| *Glycine_max* | Glyma.07G071000 | Glycine_max­_EGL1_GL3_lik_a | Phytozome |
| *Glycine_max* | Glyma.03G009500 | Glycine_max_EGL1_GL3_like_b | Phytozome |
| *Glycine_max* | Glyma.05G208300 | Glycine_max_EGL1_GL3_like_c | Phytozome |
| *Glycine_max* | Glyma.08G014900 | Glycine_max_EGL1_GL3_like_d | Phytozome |
| *Gossypium_raimondii* | Gorai.004G212200 | Gossypium_raimondii_EGL1_GL3_like_ | Phytozome |
| *Kalanchoe_dp* | 0101s0080 | Kalanchoe_dp_EGL1_GL3_like_ | Phytozome |
| *Kalanchoe_dp* | 0472s0024 | Kalanchoe_dp_EGL1_GL3_like_ | Phytozome |
| *Kalanchoe_laxiflora* | Kalax.0262s0008 | Kalanchoe_laxiflora_EGL1_GL3_like_ | Phytozome |
| *Kalanchoe_laxiflora* | Kalax.1257s0001 | Kalanchoe_laxiflora_EGL1_GL3_like_ | Phytozome |
| *Manihot_esculenta* | Manes.02G019400 | Manihot_esculenta_EGL1_GL3_like_ | Phytozome |
| *Manihot_esculenta* | Manes.05G034700 | Manihot_esculenta_EGL1_GL3_like_ | Phytozome |
| *Medicago_truncatula* | Medtr8g098275 | Medicago_truncatula_EGL1_GL3_like_ | Phytozome |
| *Mimulus_guttatus* | Migut.E01090 | Mimulus_guttatus_EGL1_GL3_like_ | Phytozome |
| *Oryza_sativa* | Os01g39480 | Oryza_sativa_GL3_like_a | Phytozome |
| *Oryza_sativa_* | Os01g39580 | Oryza_sativa_GL3_like_b | Phytozome |
| *Oryza_sativa_* | Os04g47040 | Oryza_sativa_GL3_like_c | Phytozome |
| *Oryza_sativa_* | Os04g47059 | Oryza_sativa_GL3_like_d | Phytozome |
| *Oryza_sativa_* | Os04g47080 | Oryza_sativa_GL3_like_e | Phytozome |
| *Oryza_sativa_* | Os11g15210 | Oryza_sativa_GL3_like_f | Phytozome |
| *Panicum_hallii_* | Pahal.G01894 |  | Phytozome |
| *Panicum_virgatum_* | Pavir.Gb01093 |  | Phytozome |
| *Phaseolus_vulgaris* | Phvul.002G290000 | Phaseolus_vulgaris_EGL1_GL3_like_Phvul.002G290000 | Phytozome |
| *Phaseolus_vulgaris* | Phvul.010G098500 | Phaseolus_vulgaris_EGL1_GL3_like_Phvul.010G098500 | Phytozome |
| *Populus_trichocarpa* | Potri.001G103600 | Populus_trichocarpa_EGL1_GL3_like_Potri.001G103600 | Phytozome |
| *Populus_trichocarpa* | Potri.002G159400 | Populus_trichocarpa_EGL1_GL3_like_Potri.002G159400 | Phytozome |
| *Populus_trichocarpa* | Potri.003G128000 | Populus_trichocarpa_EGL1_GL3_like_Potri.003G128000 | Phytozome |
| *Populus_trichocarpa* | Potri.014G083900 | Populus_trichocarpa_EGL1_GL3_like_Potri.014G083900 | Phytozome |
| *Prunus_persica* | Prupe.2G170100 | Prunus_persica_EGL1_GL3_like_Prupe.2G170100 | Phytozome |
| *Prunus_persica* | Prupe.5G100700 | Prunus_persica_EGL1_GL3_like_Prupe.5G100700 | Phytozome |
| *Ricinus_communis* | 0174.t000426 | Ricinus_communis_EGL1_GL3_like3_0174.t000426 | Phytozome |
| *Ricinus_communis* | 30190.t000459 | Ricinus_communis_EGL1_GL3_like_30190.t000459 | Phytozome |
| *Salix_purpurea* | SapurV1A.0046s0390 | Salix_purpurea_EGL1_GL3_like_SapurV1A.0046s0390 | Phytozome |
| *Salix_purpurea* | SapurV1A.0098s0030 | Salix_purpurea_EGL1_GL3_like_SapurV1A.0098s0030 | Phytozome |
| *Salix_purpurea* | SapurV1A.0139s0380 | Salix_purpurea_EGL1_GL3_like_SapurV1A.0139s0380 | Phytozome |
| *Salix_purpurea* | SapurV1A.0612s0160 | Salix_purpurea_EGL1_GL3_like_SapurV1A.0612s0160 | Phytozome |
| *Saruma_henryi* | DN19834_c2_g3_i1 | Saruma_henryi_GL3_like_DN19834_c2_g3_i1 | Evo Devo Transcriptomes |
| *Saruma_henryi* | DN19834_c2_g3_i2 | Saruma_henryi_GL3_like_DN19834_c2_g3_i2 | Evo Devo Transcriptomes |
| *Setaria_italica* | Seita.5G411500 | Setaria_italica_Seita.5G411500 | Phytozome |
| *Setaria_italica* | Seita.7G195400 | Setaria_italica_Seita.7G195400 | Phytozome |
| *Setaria_viridis* | Sevir.7G207500 | Setaria_viridis_Sevir.7G207500 | Phytozome |
| *Solanum_lycopersicum* | Solyc08g081140 | Solanum_lycopersicum­_EGL1_GL3_like_Solyc08g081140 | Phytozome |
| *Sorghum_bicolor* | Sobic.006G076900 | Sorghum_bicolor_Sobic.006G076900 | Phytozome |
| *Sorghum_bicolor* | Sobic.006G175200 | Sorghum_bicolor_Sobic.006G175200 | Phytozome |
| *Sorghum_bicolor* | Sobic.006G175700 | Sorghum_bicolor_Sobic.006G175700 | Phytozome |
| *Theobroma_cacao* | Thecc1EG016261 | Theobroma_cacao_EGL1_GL3_like_Thecc1EG016261 | Phytozome |
| *Thottea_siliquosa* | DN16685_c3_g1_i22 | Thottea_siliquosa_GL3_like_DN16685_c3_g1_i22 | Evo Devo Transcriptomes |
| *Thottea_siliquosa* | DN16685_c3_g2_i3 | Thottea_siliquosa_GL3_like_DN16685_c3_g2_i3 | Evo Devo Transcriptomes |
| *Trifolium_pratense* | Tp57577 | Trifolium_pratense_EGL1_GL3_like_Tp57577 | Phytozome |
| *Vitis_vinifera* | GSVIVG01019750001 | Vitis_vinifera_EGL1_GL3_like_GSVIVG01019750001 | Phytozome |
| *Vitis_vinifera* | GSVIVG01026927001 | Vitis_vinifera_EGL1_GL3_like_GSVIVG01026927001 | Phytozome |
| *Zea_mays* | GRMZM5G822829_EGL1like | Zea_mays_GRMZM5G822829_EGL1like | Phytozome |
| *Zea_mays* |  | Zea_mays_R1 | Phytozome |
| *Zea_mays* | Zm00008a038470EGL1like | Zea_mays_Zm00008a038470EGL1like | Phytozome |
| *Zea_mays* | Zm00008a038470_EGL1like | Zea_mays_Zm00008a038470_EGL1like | Phytozome |
