# Supplementary material for: Evolution of the Subgroup 6 R2R3-MYB Genes and Their Contribution to Floral Color in the Perianth-Bearing Piperales
Source: Front Plant Sci. 2021 Apr 9;12:633227. doi: 10.3389/fpls.2021.633227 (PMC8063865; doi:10.3389/fpls.2021.633227)
Supplement: Supplementary Table 6 — Names, abbreviations, codes, and original databases for all sequences included in the WD40 phylogenetic analyses. [file Table_6.DOCX]

**Supplementary Table 6.** Names, abbreviations and codes for all sequences included in the WD40 phylogenetic analysis. In purple are all new sequences isolated in this work.

| **Species** | **Original Code** | **Assigned name in the tree** | **Database** |
| --- | --- | --- | --- |
| *Amborella_trichopoda* | AmtrTTG1 | Amborella_trichopoda_TTG1 | Phytozome |
| *Aquilegia_coerulea* | Aqcoe1G412500 | Aquilegia_coerulea_TTG1_Aqcoe1G412500 | Phytozome |
| *Arabidopsis_halleri* | Araha.0138s0001 | Arabidopsis_halleri_TTG1_Araha.0138s0001 | Phytozome |
| *Arabidopsis_lyrata* | AL6G36110 | Arabidopsis_lyrata_TTG1_AL6G36110 | Phytozome |
| *Arabidopsis_thaliana* | At5g24520 | Arabidopsis_thaliana_TTG1_At5g24520 | Phytozome |
| *Aristolochia_arborea* | DN55268_c1_g1_i3 | Aristolochia_arborea_AarTTG1_DN55268_c1_g1_i3 | Evo Devo Transcriptomes |
| *Aristolochia_clematitis* | DN9770_c3_g1_i3 | Aristolochia_clematitis_AcleTTG1_DN9770_c3_g1_i3 | Evo Devo Transcriptomes |
| *Aristolochia_deltantha* | DN18193_c2_g7_i1 | Aristolochia_deltantha_AdelTTG1_DN18193_c2_g7_i1 | Evo Devo Transcriptomes |
| *Aristolochia_fimbriata* | DN11667_c3_g3_i2 | Aristolochia_fimbriata_AfimTTG1_DN11667_c3_g3_i2 | Evo Devo Transcriptomes |
| *Aristolochia_macrophylla* | DN16551_c0_g2_i1 | Aristolochia_macrophylla_AmacTTG1_DN16551_c0_g2_i1 | Evo Devo Transcriptomes |
| *Aristolochia_praevenosa* | DN20875_c2_g3_i1 | Aristolochia_praevenosa_ApraTTG1_DN20875_c2_g3_i1 | Evo Devo Transcriptomes |
| *Aristolochia_ringens* | DN16182_c2_g1_i1 | Aristolochia_ringens_ArinTTG1_DN16182_c2_g1_i1 | Evo Devo Transcriptomes |
| *Asarum_canadense* | DN20016_c2_g3_i1 | Asarum_canadense_AcanTTG1_DN20016_c2_g3_i1 | Evo Devo Transcriptomes |
| *Asarum_europaeum* | DN2725_c0_g1_i1 | Asarum_europaeum_AeurTTG1_DN2725_c0_g1_i1 | Evo Devo Transcriptomes |
| *Brachypodium_stacei* | Brast04G103200 | Brachypodium_stacei_TTG1_Brast04G103200 | Phytozome |
| *Brassica_oleracea* | Bol022420 | Brassica_oleracea_TTG1_Bol022420 | Phytozome |
| *Brassica_rapa* | Brara.F02668 | Brassica_rapa_TTG1_Brara.F02668 | Phytozome |
| *Capsella_rubella* | Carubv10001339 | Capsella_rubella_TTG1_Carubv10001339 | Phytozome |
| *Carica_papaya* | evm.TU.supercontig_3.159 | Carica_papaya_TTG1_evm.TU.supercontig_3.159 | Phytozome |
| *Citrus_clementina* | Ciclev10005375m | Citrus_clementina_TTG1_Ciclev10005375m | Phytozome |
| *Daucus_carota* | DCAR_020377 | Daucus_carota_TTG1_DCAR_020377 | Phytozome |
| *Eutrema_salsugineum* | Thhalv10004515 | Eutrema_salsugineum_TTG1_Thhalv10004515 | Phytozome |
| *Fragaria_vesca* | gene12450 | Fragaria_vesca_TTG1_gene12450 | Phytozome |
| *Glycine_max* | Glyma.06G136900 | Glycine_max_TTG1_Glyma.06G136900 | Phytozome |
| *Kalanchoe_dp* | Kaladp0008s0695 | Kalanchoe_dp_Kaladp0008s0695 | Phytozome |
| *Kalanchoe_laxiflora* | Kalax.0140s0046 | Kalanchoe_laxiflora_Kalax.0140s0046 | Phytozome |
| *Mimulus_guttatus* | Migut.F00147 | Mimulus_guttatus_TTG1_Migut.F00147 | Phytozome |
| *Panicum_hallii* | Pahal.A02930 | Panicum_hallii_TTG1_Pahal.A02930 | Phytozome |
| *Panicum_virgatum* | Pavir.Ab02709 | Panicum_virgatum_TTG1_Pavir.Ab02709 | Phytozome |
| *Phaseolus_vulgaris* | Phvul.009G044700 | Phaseolus_vulgaris_TTG1_Phvul.009G044700 | Phytozome |
| *Populus_trichocarpa* | Potri.015G002600 | Populus_trichocarpa_TTG1_Potri.015G002600 | Phytozome |
| *Ricinum_communis* | 29428.t000006 | Ricinum_communis_TTG1_29428.t000006 | Phytozome |
| *Salix_purpurea* | SapurV1A.0326s0450 | Salix_purpurea_TTG1_SapurV1A.0326s0450 | Phytozome |
| *Salix_purpurea* | SapurV1A.0662s0190 | Salix_purpurea_TTG1_SapurV1A.0662s0190 | Phytozome |
| *Saruma_henryi* | DN17550_c0_g1_i1 | Saruma_henryi_ShenTTG1_DN17550_c0_g1_i1 | Evo Devo Transcriptomes |
| *Setaria_viridis* | Sevir.1G278700 | Setaria_viridis_TTG1_Sevir.1G278700 | Phytozome |
| *Solanum_lycopersicum* | Solyc03g081210.1 | Solanum_lycopersicum_Solyc03g081210.1 | Phytozome |
| *Solanum_tuberosum* | PGSC0003DMG400000561 | Solanum_tuberosum_TTG1_PGSC0003DMG400000561 | Phytozome |
| *Solanum_tuberosum* | PGSC0003DMG400026477 | Solanum_tuberosum_TTG1_PGSC0003DMG400026477 | Phytozome |
| *Solanum_tuberosum* | PGSC0003DMG400044039 | Solanum_tuberosum_TTG1_PGSC0003DMG400044039 | Phytozome |
| *Theobroma_cacao* | Thecc1EG015520 | Theobroma_cacao_TTG1_Thecc1EG015520 | Phytozome |
| *Thottea_siliquosa* | DN18110_c0_g1_i1 | Thottea_siliquosa_ThsiTTG1_DN18110_c0_g1_i1 | Evo Devo Transcriptomes |
| *Trifolium_pratense* | Tp57577 | Trifolium_pratense_TTG1_Tp57577 | Phytozome |
| *Zea_mays* | GRMZM2G058292 | Zea_mays_TTG1_GRMZM2G058292 | Phytozome |
